# Seasonal shifts in vegetation, soil properties, and microbial communities in Western Himalayan forests

**DOI:** 10.1186/s40793-025-00842-y

**Published:** 2026-01-06

**Authors:** Huma Ali, Muhammad Rafiq, Muhammad Manzoor, Syed Waseem Gillani, Allan Degen, Awais Iqbal, Wenyin Wang, Muhammad Khalid Rafiq, Zhanhuan Shang

**Affiliations:** 1 School of Life Sciences, Lanzhou University, Lanzhou, 730000 China; 2 State Key Laboratory of Herbage Improvement and Grassland Agro- ecosystems, College of Ecology, Lanzhou University, Lanzhou, 730000 China; 3 Department of Microbiology, Engineering and Management Sciences, Balochistan University of Information Technology, Quetta, Pakistan; 4 Department of Plant Sciences, Quaid-i-Azam University, Islamabad, Pakistan; 5 Desert Animal Adaptations and Husbandry, Wyler Department of Dryland Agriculture, Blaustein Institutes for Desert Research, Ben-Gurion University of the Negev, Beer Sheva, 8410500 Israel; 6 Rangeland Research Institute, National Agricultural Research Center, Islamabad, 44000 Pakistan

**Keywords:** Forests, Himalaya, Metagenome, Seasons, Soil microbial diversity, Vegetation

## Abstract

**Background:**

The western Himalayan forest ecosystem faces escalating pressures from climate change and anthropogenic activities, demanding improved conservation strategies. Effective management requires understanding the seasonal fluctuations in vegetation, soil properties and microbial communities, but they remain poorly characterized across high altitude forests. We assessed these variables in 10 forest sites during the winter of 2023 and summer of 2024, analysing vegetation diversity, soil parameters, and microbial metagenomics.

**Results:**

We found pronounced seasonal shifts in plant and microbial diversities, and in soil properties. Plant species richness, and Shannon and Simpson diversity indices were higher (*p* < 0.001) in summer than in winter while the community maturity index was higher (*p* < 0.02) in winter than in summer. Soil properties exhibited clear seasonal patterns: pH, available phosphorus (AP), microbial biomass carbon (MBC) and cation exchange capacity (CEC) were higher (*p* < 0.05) in summer, whereas soil moisture (SM) and soil organic carbon (SOC) were higher (*p* < 0.05) in winter. Microbial alpha diversity indices (Shannon, Chao, and Sobs) were elevated (*p* < 0.05) in summer, while the Simpson index was elevated in winter, indicating a shift in community dominance. Beta diversity analyses revealed a significant seasonal shift in overall metabolic potential (KEGG orthologs; ANOSIM *R* = 0.222, *p* = 0.016), but not in general protein functions (COG), carbohydrate-active enzymes (CAZy), or taxonomic composition (RefSeq). Therefore, despite taxonomic turnover, core metabolic functions were maintained, indicating strong functional redundancy. Structural equation models (SEM) confirmed distinct seasonal dynamics, revealing stronger plant-soil-microbe interactions and a greater proportion of variance explained by the model in summer (R^2^=0.64–0.72 for key paths) than in winter (R^2^=0.52–0.63).

**Conclusions:**

The findings demonstrate that the western Himalayan ecosystem undergoes a fundamental seasonal reorganization. Summer is characterized by increased biodiversity, distinct soil conditions, and more dynamic microbial-ecosystem interactions, while winter exhibits greater community maturity and functional stability. The resilience of core ecosystem processes is underpinned by microbial functional redundancy, which ensures metabolic continuity despite taxonomic shifts. We recommend that forest management strategies account for these seasonal dynamics and focus on preserving the conditions that support this critical functional redundancy.

**Supplementary Information:**

The online version contains supplementary material available at 10.1186/s40793-025-00842-y.

## Background

The western Himalayan forests of Azad Jammu and Kashmir (AJK), Pakistan, provide a wide range of ecological services and support the livelihoods of many local inhabitants [1]. These forests are characterized by diverse geographical and climatic conditions, supporting multiple forest sites, each dominated by specific tree species [2]. Seasonal environmental variations influence the ecological dynamics of these ecosystems [3], as changes in temperature and precipitation patterns affect vegetation growth, soil properties, and microbial diversity. For example, snowfall plays a key role in maintaining soil moisture and forest health in the Himalayas, and its decline can lead to increased dryness and shifts in species composition [4].

Research in other extreme environments, such as alpine tundra and boreal forests, demonstrated that soil microbial communities exhibited remarkable resilience to seasonal fluctuations. Studies in these systems demonstrated that while microbial taxonomy shifted with season, core ecosystem functions were often maintained through mechanisms such as functional redundancy [5]. However, the Himalayan region, with its unique combination of extreme altitudinal gradients, specific biogeographic history, and distinct forest sites, remains a critical test case for these theories. It is unclear whether the patterns observed in boreal or alpine tundra systems occur in a biodiverse, high-altitude forest matrix where plant community composition is more complex.

Previous studies highlighted the ecological importance of these forests in maintaining biodiversity [6], regulating hydrological cycles, and providing habitats for diverse plant and animal species. Forests dominated by *Abies pindrow*, *Cedrus deodara*, and *Pinus wallichiana* are crucial for supporting local livelihoods through timber and non-timber forest products. However, these ecosystems are vulnerable to climate change, deforestation, and overgrazing, which threaten their sustainability and the services they provide.

Understanding the natural seasonal dynamics of vegetation, soil properties, and microbial diversity in the western Himalayan forests is essential for developing effective conservation and sustainable management strategies, thus ensuring the long-term health of these ecosystems [7]. These forests are biodiversity hotspots and provide vital ecosystem services such as carbon sequestration, water regulation, and nutrient cycling [8, 9]. Key soil parameters, including pH, microbial biomass carbon (MBC) and soil organic carbon (SOC), are critical indicators of soil fertility and ecosystem productivity, with seasonal variations influencing microbial activity and nutrient availability. These parameters are part of a dynamic plant-soil-microbe feedback loop that governs ecosystem multifunctionality. However, most studies in this region have focused on either vegetation or soil properties in isolation, neglecting the role of soil microbes. Furthermore, while the concept of functional redundancy is established in ecology, empirical evidence demonstrating its role in stabilizing ecosystem processes across seasons in a topographically complex and biodiverse region like the western Himalayas is scarce. This gap is critical because, as our findings demonstrated, microbial functional diversity is the key mechanism stabilizing ecosystem processes between seasons. Therefore, effective management for multifunctionality should not focus on plants or soil alone, but must target the preservation of the soil habitat, specifically organic matter content and minimal physical disruption, that supports the diverse and functionally redundant microbial communities essential for long-term resilience. Additionally, soil moisture plays a pivotal role in influencing microbial communities and plant growth, and is particularly important in mountainous regions where water availability fluctuates with altitude and precipitation patterns.

Recent advances in metagenomics have enabled insights into microbial diversity and functional potential. Alpha diversity metrics indicate microbial richness within specific niches, while beta diversity compares community shifts between seasons. To our knowledge, this is the first study to integrate seasonal metagenomics, vegetation dynamics, and soil biogeochemistry across western Himalayan forest sites, thereby directly testing how the theoretical framework of microbial functional resilience, developed in other biomes, applies to this understudied and critical region. By employing structural equation models (SEM), we provide novel insights into the strength of plant-soil-microbe interactions between seasons, a critical yet overlooked dimension in high-altitude forest management.

The western Himalayan ecosystem is subjected to strong seasonal resource fluctuations, with summer providing favourable conditions for growth and winter imposing physiological stresses. We therefore hypothesized that seasonal shifts in climate drive a coordinated reorganization of the plant-soil-microbe system, where summer conditions promote high plant and microbial diversities and activities, thereby enhancing ecosystem processes, while winter conditions favour a more mature plant community and the accumulation of soil organic matter. Specifically, we predicted that in summer compared to winter: (1) plant species richness and diversity, and microbial alpha-diversity would be higher; (2) microbial biomass carbon and nutrient-cycling activity would be elevated; while (3) soil moisture and organic carbon would be lower due to increased microbial decomposition and plant uptake. We further hypothesized that despite these seasonal shifts in taxonomy and activity, microbial functional redundancy would maintain the stability of core ecosystem processes across both seasons and that the strength of these relationships would be distinct from patterns observed in other well-studied biomes due to the unique selective pressures of the Himalayan environment.

## Materials and methods

### Study area

**Fig. 1:**
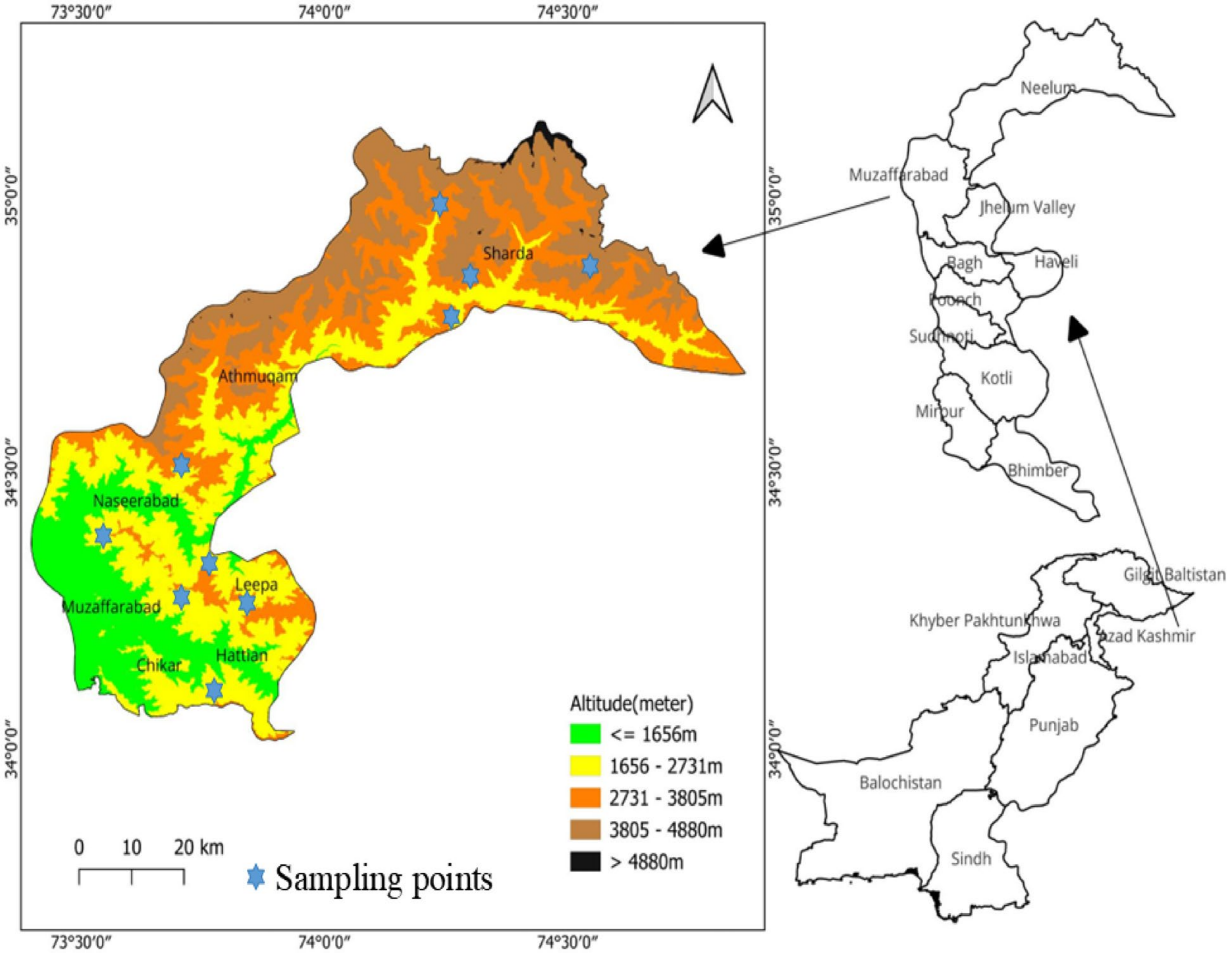
Map of the study area depicting ten forest sites

This study was conducted in the Neelum, Jhelum, and Muzaffarabad valleys in the western Himalayan region of Azad Jammu and Kashmir (AJK), Pakistan, in the winter of 2023 and summer of 2024. The site is characterized by a temperate climate, with mean annual temperatures of 15–20 °C [10], and mean annual precipitation of 1200–1500 mm [11]. Cold, snowy winters and warm, rainy summers influence the vegetation development, soil properties, and microbial diversity [12]. The study employed a paired design, with the same ten forest sites (34°5’19.7088” N to 34°56’2.616” N latitude; 74°13’26.4” E to 73°51’28.08” E longitude) sampled in winter and summer. This approach inherently controls inter-annual variation by focusing on the relative seasonal differences within each site, which are expected to be robust across years given the magnitude of the region’s seasonal climatic shift. The altitude of the ten forest sites ranged between 1,021 and 2,816 m above sea level (Fig. 1).

### Vegetation survey and measurements

A plot of one ha (100 m × 100 m) was established in each forest site. Within each plot, five random 10 m × 10 m sub-plots were selected, with a minimum distance of > 20 m between sub-plot centres to ensure spatial independence. Within each sub-plot, a 1 m × 1 m quadrat was used to quantify herbaceous species. Density, cover, and frequency of each tree and shrub species were measured within the sub-plots.

Plant species were identified based on morphological characteristics (Supplementary Tables S1–S20). The Shannon-Wiener index (H′) was used to determine species diversity, Simpson’s index (D) for dominance, Pielou’s evenness index (J) for species distribution uniformity, and species richness (SR) for species abundance.

Shannon diversity index was calculated as:

1 $$ H^{\prime } = - \sum {pi\log pi} $$

where: Pi = ni/N, N=∑ni = total number of individuals of all species; ni = number of individuals of one species.

Simpson diversity index was calculated as:

2 $$ D = \frac{{\sum {ni(ni - 1)} }}{{N(N - 1)}} $$

where ni = number of individual species; N = total number of individuals of all species.

Species evenness was calculated as [13]:

3 $$J = \frac{H'}{{LogS}} $$

where H' = Shannon’s diversity index; S = total number of species in a community.

Species richness was calculated as [14]:

4 $$ SR = \frac{S}{{\sqrt N }} $$

where S = total number of species; N = total number of individuals.

Species maturity was determined by the method of [15]:

5 $$ Species\:Maturity = \frac{F}{S} $$

where F = total frequency of a community; S = total species of a community.

### Soil sampling and analyses

Five replicate soil samples were collected from each of two soil depths, 0–10 cm and 10–20 cm, in each sub-plot using a soil auger of 12.6 cm diameter (Eijkelkamp, Giesbeek, Netherlands), stored in polyethylene bags and transported to the laboratory. A total of 200 soil samples were collected for physicochemical analysis, five samples at two depths from 10 forest sites in both winter and summer.

Soil samples were air-dried, ground, and passed through a 2-mm sieve. Physical measurements included soil electric conductivity (EC), pH, soil texture, soil moisture content (SMC), and bulk density (BD). Chemical analyses included soil organic carbon (SOC), total phosphorus (TP), total potassium (TK), total nitrogen (TN), available phosphorus (AP), potassium (AK), cation exchange capacity (CEC), and microelements, including iron (Fe), copper (Cu), manganese (Mn) and zinc (Zn). To obtain a representative metagenomic profile for each site and to manage sequencing costs, soil samples from the upper soil depth at each location were composited. This resulted in a total of 20 composite samples (10 forest sites × 2 seasons), which were used to determine seasonal shifts in microbial communities across the surveyed forests of the Western Himalayas. A portion of each sample was stored at -20˚C for soil microbial biomass carbon (MBC) analysis, while another portion was stored at -80˚C for metagenomic analysis (Majorbio Company, Shanghai, China). Microbial biomass carbon (MBC) and soil physicochemical properties were analysed according to methods detailed in the Supplementary File. Metagenomic DNA was extracted and sequenced as follows [16].

### Metagenomics analysis

#### DNA extraction and quality control

Genomic DNA was extracted from soil samples using the DNeasy PowerSoil Kit (Qiagen, Hilden, Germany), and DNA integrity and quality were assessed via 1% agarose gel electrophoresis. The DNA was fragmented to ~ 350 bp using a ultrasonicator (Covaris M220, Woburn, MA, USA). Paired-end (PE) libraries were constructed by ligating a “Y” adapter, followed by magnetic bead screening to remove autolinkers. PCR amplification enriched the library templates, and denaturation with sodium hydroxide yielded single-stranded DNA for sequencing.

The library was amplified by bridge PCR to generate DNA clusters, followed by Illumina sequencing using fluorescent dNTPs and modified polymerase. Each cycle incorporated single bases, detected via laser scanning before fluorophore cleavage. Sample-specific index tags facilitated multiplexing, and raw FASTQ data were quality-filtered using fastp (https://github.com/OpenGene/fastp, v0.20.1) [17], discarding low-quality reads (Q < 20, length < 50 bp) and adapter sequences. High-quality clean data were retained for downstream analysis (Supplementary Tables S21–S30, Figures S1, S2). Sequencing depth and coverage were assessed using nonpareil v3.5.5 [18]. Paired-end reads were processed on the Galaxy platform to estimate average coverage and the resulting data were visualized in R v.4.5.1 to generate coverage curves (Fig. S3).

#### Assembly and gene prediction

Clean reads were assembled into contigs using MEGAHIT (v1.1.2) [19], retaining contigs ≥ 300 bp. Open reading frames (ORFs) were predicted using Prodigal (https://github.com/hyattpd/Prodigal, v2.6.3) [20], with ORFs ≥ 100 bp translated into amino acid sequences. ORFs from all samples were clustered using CD-HIT (http://www.bioinformatics.org/cd-hit/, v4.6.1) [21] with identity and coverage thresholds of 90% to construct a non-redundant gene set. The longest sequence in each cluster was selected as the representative.

#### Taxonomic and functional annotation

To determine microbial community composition, the clean reads from each sample were mapped back to the NR gene catalog using SOAPaligner (version 2.21) [21] with a 95% identity threshold. The abundance of each gene in a sample was calculated based on the read count. For taxonomic annotation, the non-redundant gene set was aligned against the NCBI non-redundant protein database (RefSeq NR) using DIAMOND (https://github.com/bbuchfink/diamond, blastp, E-value ≤ 1e − 5) [22]. This is a comprehensive database of amino acid sequences of non-redundant proteins, including SwissProt, Protein Information Resource (PIR), Protein Research Foundation (PRF), non-redundant data from Protein Data Bank (PDB) protein databases, and protein data translated from GenBank and RefSeq’s CDS data. The taxonomic lineage for each gene was assigned based on the lowest common ancestor (LCA) of the best hits. For each sample, the abundance of all genes assigned to the same taxon was summed to generate species-level abundance profiles. These profiles were used for all subsequent taxonomic and alpha-diversity analyses. Taxonomic annotations were derived from the NR database, and species abundances were determined at various taxonomic levels (domain to order). Sequences were aligned to the eggNOG database using DIAMOND (https://github.com/bbuchfink/diamond, blastp, E-value ≤ 1e-5) [23] to assign clusters of orthologous groups (COGs), and to the Kyoto Encyclopedia of Genes and Genomes (KEGG) genes database (http://www.genome.jp/kegg/, blastp, E-value ≤ 1e-5) [24] to annotate pathways, modules, and orthologs (KOs). In addition, sequences were aligned to the CAZy database using hmmscan (http://www.cazy.org/, E-value ≤ 1e-5) [25] to identify carbohydrate-active enzymes (CAZymes), including glycoside hydrolases (GHs), glycosyltransferases (GTs), and polysaccharide lyases (PLs).

### Statistical analyses

All statistical analyses and data visualizations were performed in R Studio (version 4.5.1). A map of the study area was generated using QGIS software (version 3.32.0).

Sequencing depth and coverage were assessed using nonpareil (version 3.5.5) [18] and the resulting coverage data were visualized in R using the ggplot2 package [26]. Alpha diversity indices (Shannon, Chao1) for the microbial communities were calculated based on the species-level abundance profiles after all samples were rarefied to an even sequencing depth of 67,126,532 sequences per sample to ensure equitable comparisons. Differences in these microbial alpha-diversity indices between seasons were tested by the non-parametric Wilcoxon rank-sum test and results were visualized using the ggplot2 package [26].

The effects of season, soil depth, and their interactions on soil physicochemical properties were tested using a two-way analysis of variance (ANOVA). Post-hoc Tukey’s honest significant difference (HSD) test was applied for pairwise comparisons where significant effects were found. Differences in vegetation diversity metrics between seasons were also evaluated using the non-parametric Wilcoxon rank-sum test. Relationships between vegetation diversity indices were tested using Spearman’s rank correlation.

Variation in microbial community composition, beta-diversity, was visualized using principal coordinate analysis (PCoA) based on Bray-Curtis dissimilarities. The statistical significance of seasonal clustering was tested with analysis of similarities (ANOSIM) using the vegan package [27] in R Studio (version 4.5.1). The relative abundances of microbial taxa (based on non-redundant gene catalog annotations) and functional profiles (KEGG, COG, CAZy) were visualized using stacked bar plots generated with ggplot2 package [26]. To examine the seasonal dynamics of soil properties, plant diversity, microbial biomass carbon (MBC), microbial taxonomy, functional potential, alpha diversity, and ecosystem multifunctionality, we constructed separate structural equation models (SEM) for the winter and summer seasons using AMOS 20.0 software [28]. To mitigate multicollinearity among the numerous interrelated variables, including soil properties (SOC, TN, TP, TK, SM, pH), MBC, microbial alpha diversity, functional profile (KEGG, COG, CAZy), and taxonomic abundances (*Actinomycetota*, *Pseudomonadota*, *Acidobacteriota*), we employed principal component analysis (PCA) to create multivariate composite indices. The first principal component (PC1) for each conceptual group (e.g., soil properties, taxonomy) was used as a latent variable in the SEM, as each PC1 explained > 50% of the variance within its respective dataset. The models were fitted using the maximum likelihood estimation method, and the goodness-of-fit was evaluated against standard criteria, including a root mean square error of approximation (RMSEA) value of ≤ 0.08 and a comparative fit index (CFI) value of ≥ 0.90. Ecosystem multifunctionality (EMF) was calculated using the averaging approach method [29]. We integrated multiple variables representing key ecosystem dimensions: (1) soil properties (SOC, TN, TP, TK, SM, pH); (2) plant community diversity (Shannon index); (3) microbial community structure and biomass (microbial alpha-diversity Shannon index and MBC); (4) genetic functional potential (abundance of genes annotated to KEGG, COG, and CAZy databases); and (5) microbial taxonomy (relative abundance at the phylum level). Each variable was first normalized to a 0–1 scale based on observed minimum and maximum values across all samples. The EMF index for each sample was then derived as the arithmetic mean of all normalized values and this integrated index was used as a distinct response variable in subsequent analyses.

### Accession numbers

The raw metagenomic sequencing data in this study are deposited in the China National Centre for Bioinformation (CNCB) under Bio Project accession number CRA027991. The data are accessible publicly via the CNCB repository (https://ngdc.cncb.ac.cn/gsa/browse/CRA027991).

## Results

### Seasonal variations in vegetation and diversity

Species richness (1.98 vs. 1.21), Shannon diversity index (3.20 vs. 2.57), a measure of community diversity considering richness and evenness, and Simpson diversity index (0.94 vs. 0.87), a measure of dominance, were all higher (all *p* < 0.01) in summer than in winter.

**Fig. 2:**
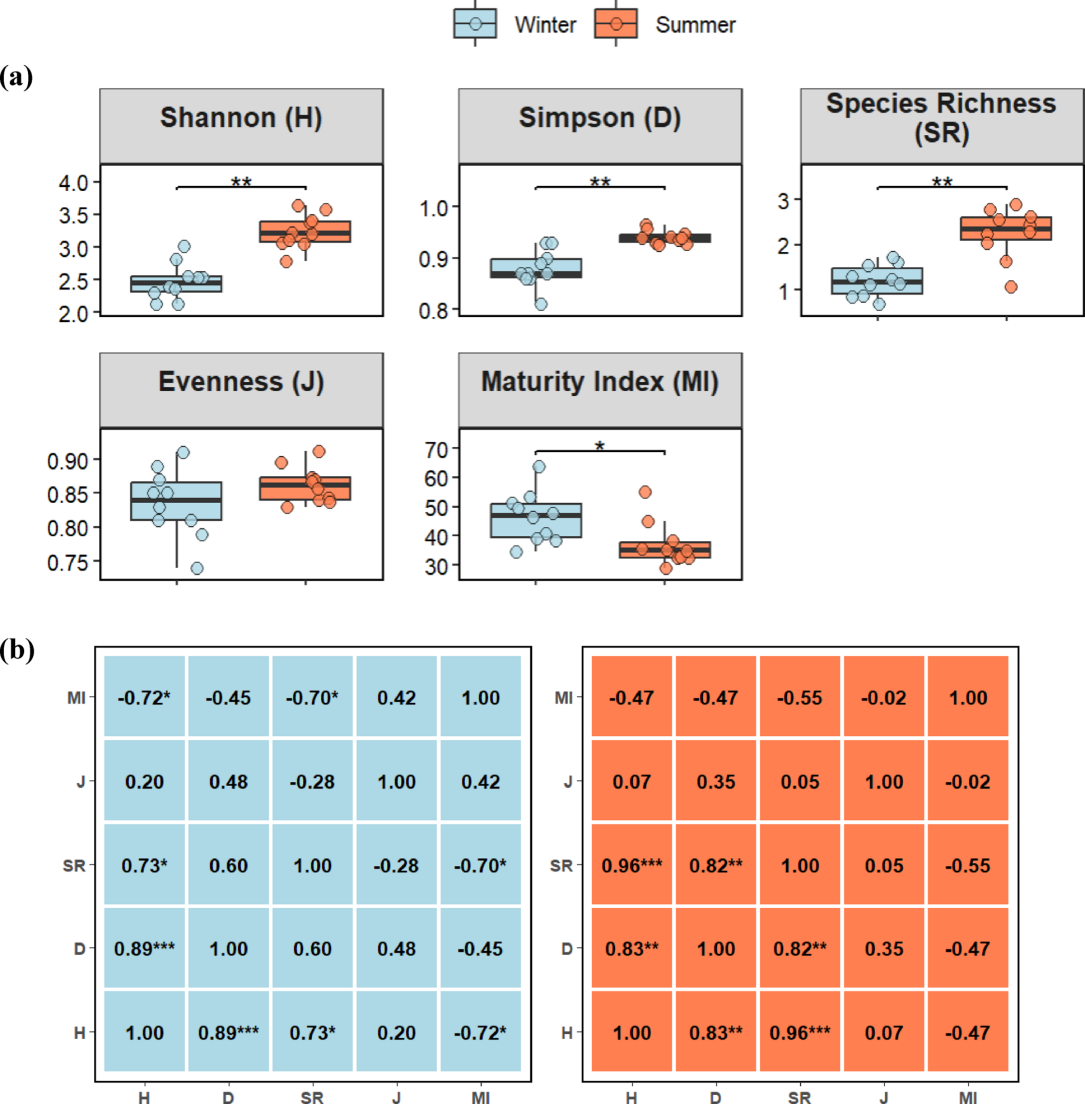
**a** Box plots displaying seasonal differences in plant diversity indices between winter and summer. Comparisons were assessed by applying the non-parametric Wilcoxon rank-sum test with Benjamini-Hochberg adjustment; *p < 0.05, **p < 0.01, ***p < 0.001. **b** Spearman’s rank correlations for two seasons, Shannon diversity index (H'), Simpson diversity index (D), species richness (SR), species evenness (J), and maturity index (MI); *p < 0.05, **p < 0.01, ***p < 0.001

Pielou’s evenness, which measures the evenness of species distribution, did not differ between seasons, while the community maturity index was higher (*p* < 0.05) in winter (49.2) than in summer (42.1) (Fig. 2a).

In Spearman’s rank correlation analysis (Fig. 2b), a positive correlation emerged between Shannon diversity and Simpson diversity (*p* < 0.001) and between SR and Simpson diversity (*p* < 0.05) in winter; whereas a negative correlation (*p* < 0.05) emerged between community maturity index (MI) and SR in winter.

### Seasonal variations in soil properties

**Fig. 3:**
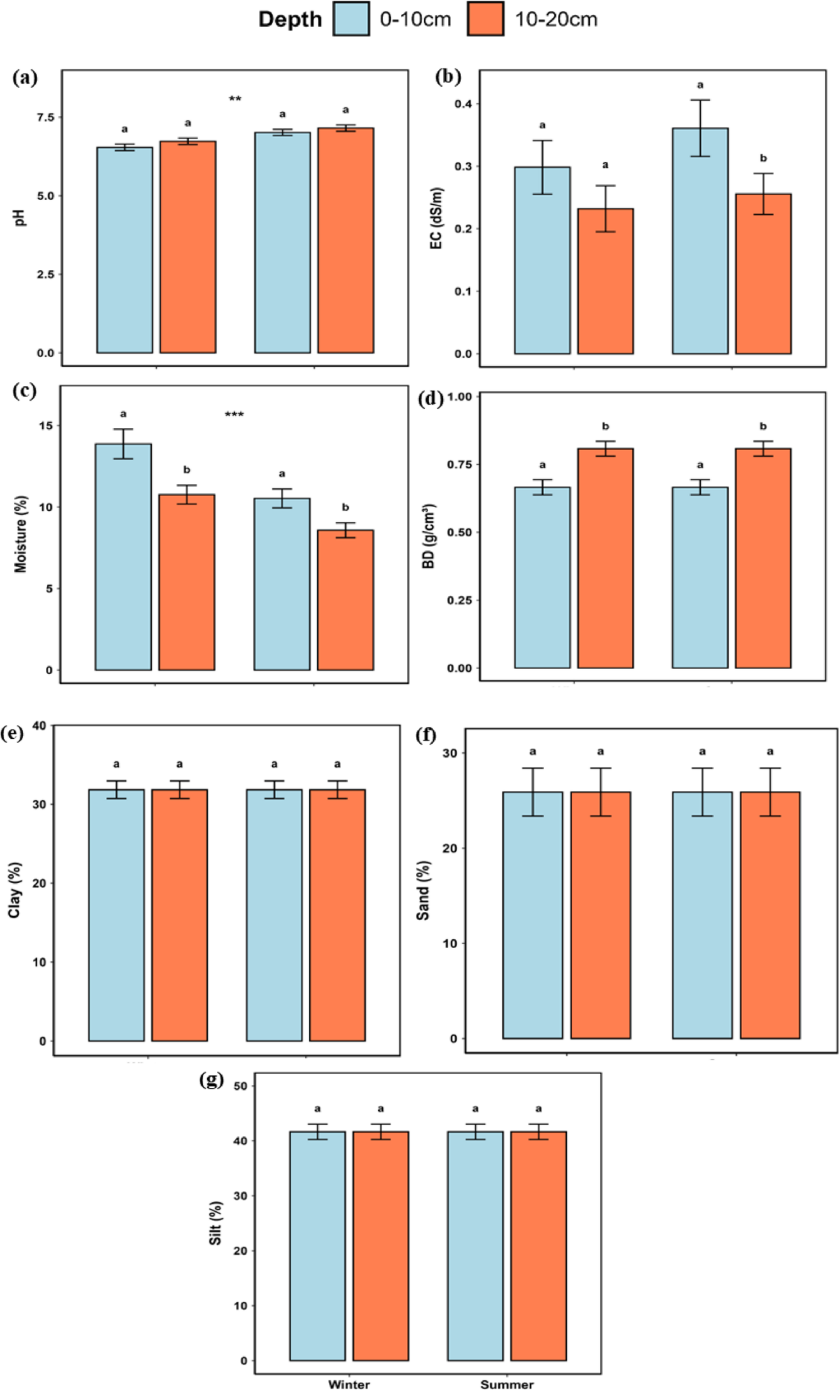
Means ± SD of **a** soil pH, **b** electric conductivity (EC, dS/m), **c** moisture content (%), **d** bulk density (BD, g/cm^3^) and **e**, **f**, **g** texture (% clay, sand and silt) in soil at 0–10 cm and 10–20 cm in winter and in summer. Means with different superscripts within seasons differ from each other (*p* < 0.05). Asterisks on shoulder lines indicate significant differences between season; *p < 0.05, **p < 0.01, ***p < 0.001

The two-way ANOVA revealed significant and distinct trends for soil properties between seasons and depths. The pH was higher (*p* < 0.001) in summer than in winter, while soil moisture decreased (*p* < 0.001) with depth and was higher (*p* < 0.001) in winter than in summer. Bulk density (BD) increased (*p* < 0.001) with depth, while electric conductivity (EC) and soil texture, comprising clay, sand, and silt did not differ (*p* > 0.05) between seasons or soil depths (Fig. 3).

**Fig. 4:**
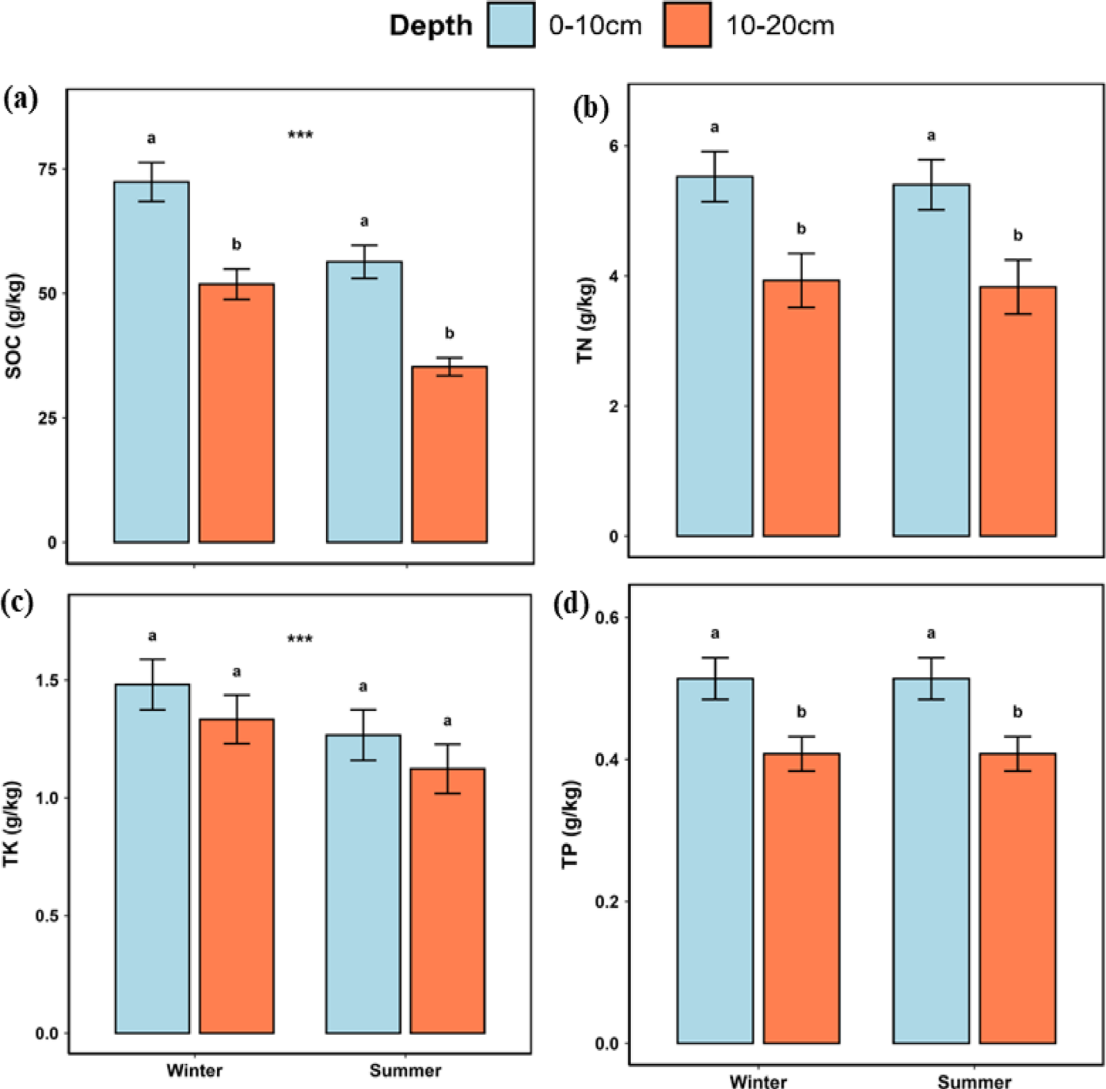
Means ± SD of **a** soil organic carbon (SOC, g/kg), **b** total nitrogen (TN, g/kg), **c** total potassium (TK, g/kg) and **d** total phosphorus (TP, g/kg) in soil at 0–10 cm and 10–20 cm in winter and in summer. Means with different superscripts within seasons differ from each other (*p* < 0.05). Asterisks on shoulder lines indicate significant differences between seasons; *p < 0.05, **p < 0.01, ***p < 0.001

Soil organic carbon (SOC) was higher (*p* < 0.001) in the topsoil than lower layer and exhibited a strong seasonal effect, being greater (*p* < 0.001) in winter than in summer. Total nitrogen (TN) decreased (*p* < 0.001) with soil depth; total potassium (TK) was higher (*p* < 0.05) in winter than in summer, while total phosphorus (TP) decreased (*p* < 0.001) with soil depth (Fig. 4).

Available potassium (AK) was higher (*p* < 0.05) in winter than in summer; while available phosphorus (AP) was higher (*p* < 0.01) in summer than in winter and decreased (*p* < 0.001) with soil depth. Microbial biomass carbon (MBC) and cation exchange capacity (CEC) were higher (*p* < 0.001) in summer than in winter (Fig. S4).

The micronutrients, iron (Fe), copper (Cu), zinc (Zn) and manganese (Mn) decreased (*p* < 0.001) with soil depth, but did not differ between seasons (Fig. S5). The interaction between season and depth was not significant for any of the variables measured (Tables 1 and 2).

**Table 1:** Two way ANOVA results for soil physical properties, pH, electric conductivity (EC; dS/m), bulk density (BD; g/cm^3^), moisture (%), clay(%), sand (%), silt (%) indicating effect of season, depth and their interaction

Soil parameters	Term	DF	Sum of Squares	Mean Square	F-value	*p*-value	Significance
pH	Season	1	6.03	6.03	19.91	< 0.001	***
	Depth	1	0.83	0.83	2.74	0.10	ns
	Interaction	1	0.02	0.02	0.07	0.79	ns
	Residual	116	35.11	0.30			
EC (dS/m)	Season	1	0.06	0.06	1.18	0.28	ns
	Depth	1	0.22	0.22	4.67	< 0.05	*
	Interaction	1	0.01	0.01	0.24	0.63	ns
	Residual	116	5.49	0.05			
BD(g/cm^3^)	Season	1	0.00	0.00	0.00	1.00	ns
	Depth	1	0.60	0.60	26.73	< 0.001	***
	Interaction	1	0.00	0.00	0.00	1.00	ns
	Residual	116	2.62	0.02			
Moisture (%)	Season	1	229.16	229.16	17.93	< 0.001	***
	Depth	1	192.31	192.31	15.05	< 0.001	***
	Interaction	1	10.05	10.05	0.79	0.38	ns
	Residual	116	1482.29	12.78			
Clay (%)	Season	1	0.00	0.00	0.00	1.00	ns
	Depth	1	0.00	0.00	0.00	1.00	ns
	Interaction	1	0.00	0.00	0.00	1.00	ns
	Residual	116	4340.44	37.42			
Sand (%)	Season	1	0.00	0.00	0.00	1.00	ns
	Depth	1	0.00	0.00	0.00	1.00	ns
	Interaction	1	0.00	0.00	0.00	1.00	ns
	Residual	116	22197.64	191.36			
Silt (%)	Season	1	0.00	0.00	0.00	1.00	ns
	Depth	1	0.00	0.00	0.00	1.00	ns
	Interaction	1	0.00	0.00	0.00	1.00	ns
	Residual	116	6806.69	58.68			

*p < 0.05, ** *p* < 0.01, ***p < 0.001; and non-significant (ns) *p* > 0.05)

**Table 2:** Two-way ANOVA results for soil chemical properties, soil organic carbon (SOC; g/kg), total nitrogen (TN; g/kg), total phosphorus (TP; g/kg), total potassium (TK; g/kg), available potassium (AK; g/kg), available phosphorus (AP; g/kg), microbial biomass carbon (MBC; g/kg), cation exchange capacity (CEC; meq/100 g) iron (Fe; mg/kg), manganese (Mn; mg/kg), zinc (Zn; mg/kg), copper (Cu; mg/kg) indicating effects of season, depth, and their interaction

Soil parameters	Term	DF	Sum of Squares	Mean Square	F-value	*p*-value	Significance
SOC (g/kg)	Season	1	7988.33	7988.33	27.46	< 0.001	***
	Depth	1	12999.49	12999.49	44.68	< 0.001	***
	Interaction	1	2.28	2.28	0.01	0.93	ns
	Residual	116	33749.43	290.94			
TN (g/kg)	Season	1	0.38	0.38	0.08	0.78	ns
	Depth	1	75.15	75.15	15.58	< 0.001	***
	Interaction	1	0.00	0.00	0.00	0.98	ns
	Residual	116	559.52	4.82			
TP (g/kg)	Season	1	0.00	0.00	0.00	1.00	ns
	Depth	1	0.34	0.34	15.48	< 0.001	***
	Interaction	1	0.00	0.00	0.00	1.00	ns
	Residual	116	2.52	0.02			
TK (g/kg)	Season	1	1.35	1.35	4.03	< 0.05	*
	Depth	1	0.64	0.64	1.90	0.17	ns
	Interaction	1	0.0002	0.0002	0.00	0.98	ns
	Residual	116	38.94	0.34			
AK (g/kg)	Season	1	0.06	0.06	6.81	< 0.05	*
	Depth	1	0.03	0.03	2.83	0.10	ns
	Interaction	1	0.00	0.00	0.01	0.94	ns
	Residual	116	1.06	0.01			
AP (g/kg)	Season	1	0.0001	0.0001	7.05	< 0.01	**
	Depth	1	0.0003	0.0003	31.41	< 0.001	***
	Interaction	1	0.00	0.00	0.09	0.77	ns
	Residual	116	0.001	0.00			
MBC (g/kg)	Season	1	3.02	3.02	76.98	< 0.001	***
	Depth	1	0.00	0.00	0.00	1.00	ns
	Interaction	1	0.00	0.00	0.00	1.00	ns
	Residual	116	4.55	0.04			
CEC (meq/100 g)	Season	1	775.43	775.43	89.70	< 0.001	***
	Depth	1	0.00	0.00	0.00	1.00	ns
	Interaction	1	0.00	0.00	0.00	1.00	ns
	Residual	116	1002.78	8.64			
Fe (mg/kg)	Season	1	0.00	0.00	0.00	1.00	ns
	Depth	1	1895576.14	1895576.14	66.61	< 0.001	***
	Interaction	1	0.00	0.00	0.00	1.00	ns
	Residual	116	3301193.96	28458.57			
Mn (mg/kg)	Season	1	0.00	0.00	0.00	1.00	ns
	Depth	1	43988.32	43988.32	3.96	< 0.05	*
	Interaction	1	0.00	0.00	0.00	1.00	ns
	Residual	116	1287918.79	11102.75			
Zn (mg/kg)	Season	1	0.00	0.00	0.00	1.00	ns
	Depth	1	646.07	646.07	11.04	< 0.01	**
	Interaction	1	0.00	0.00	0	1.00	ns
	Residual	116	6790.52	58.54			
Cu (mg/kg)	Season	1	0.00	0.00	0.00	1.00	ns
	Depth	1	850.46	850.46	24.02	< 0.001	***
	Interaction	1	0.00	0.00	0.00	1.00	ns
	Residual	116	4107.95	35.41			

*p < 0.05, ** *p* < 0.01, ***p < 0.001; and non-significant (ns) *p* > 0.05)

### Seasonal variations in microbial alpha diversity

Microbial alpha diversity exhibited consistent and significant seasonal patterns. The Shannon diversity index was higher (*p* < 0.05) in summer than in winter (6.72 vs. 5.38). A contrasting seasonal pattern was observed for the Simpson diversity index, being higher (*p* < 0.05) in winter than in summer (0.026 vs. 0.0098).

**Fig. 5:**
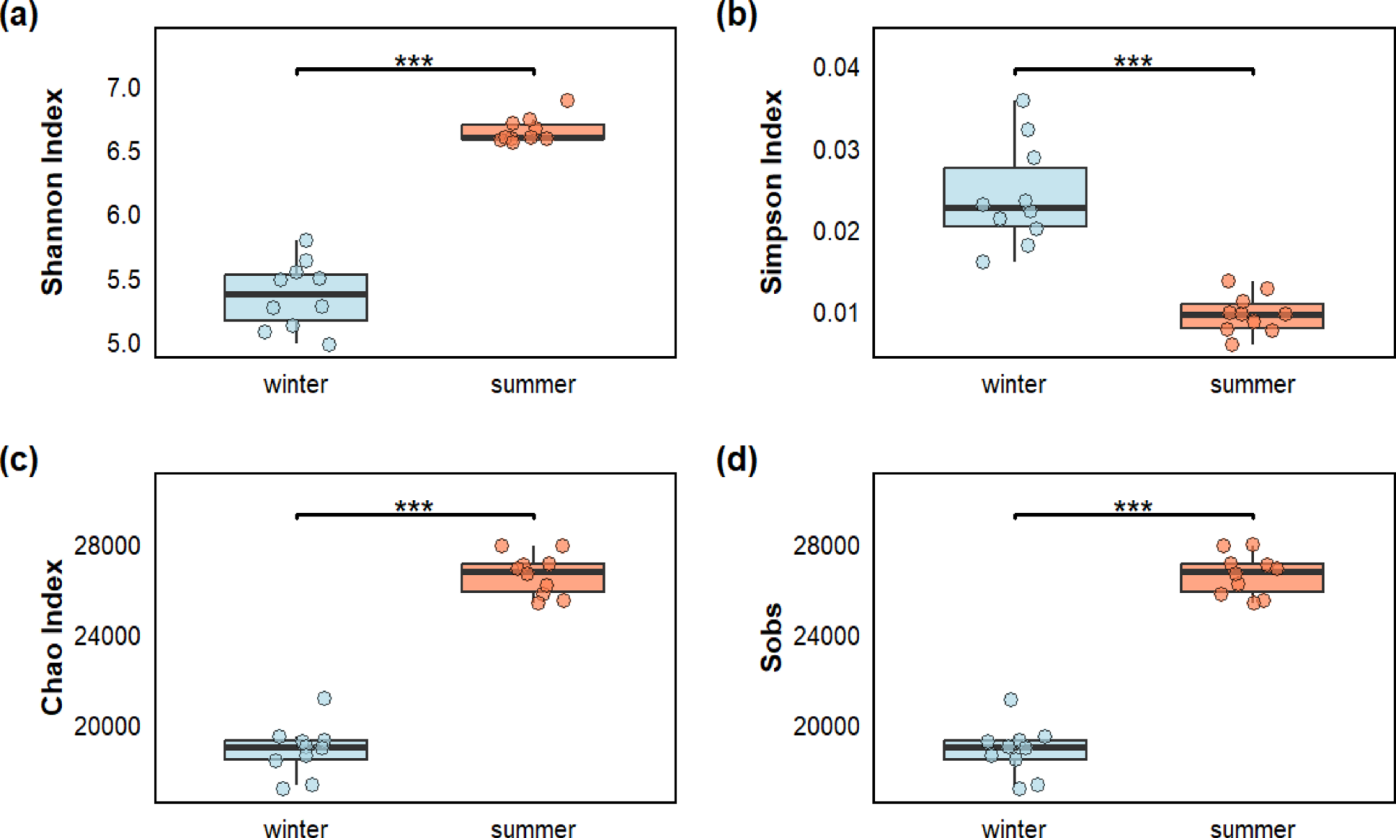
Comparison of soil microbial alpha diversity indices (**a** Shannon, **b** Simpson, **c** Chao, **d** Sobs) between seasons based on the non-parametric Wilcoxon test; ***p < 0.01

Both the Chao and the Sobs indices, which estimate species richness, were higher (*p* < 0.05) in summer than in winter. The Sobs index revealed a substantial increase in richness from winter (19074) to summer (26647), while the Chao index displayed a similar trend of elevated richness in the summer season (Fig. 5).

### Seasonal variations in taxon function beta diversity

#### COG functional beta diversity

**Fig. 6:**
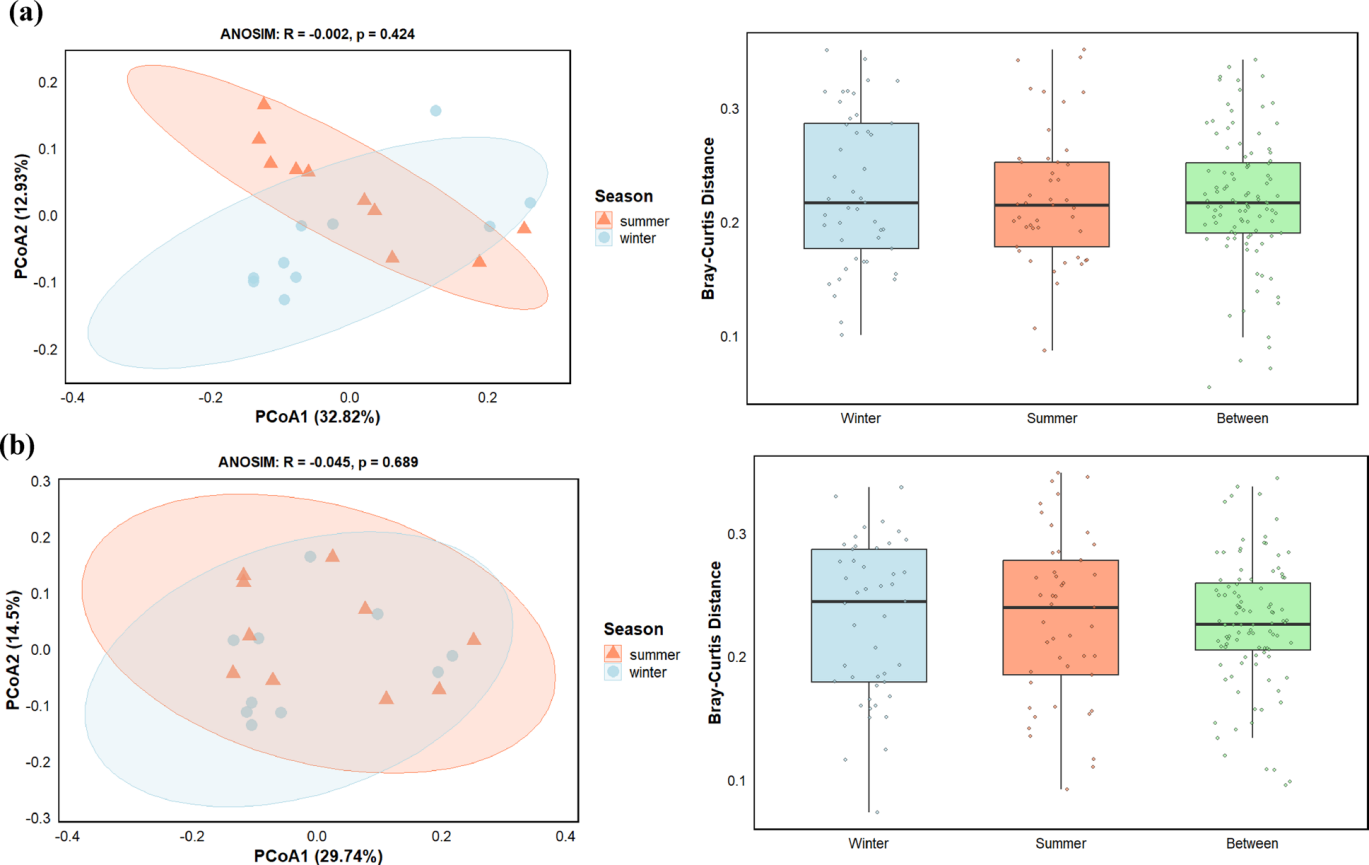
Comparison of beta diversity of microbial communities between winter and summer by Bray-Curtis dissimilarity. Plots confirm the patterns observed in the principal coordinate analysis (PCoA) for **a** clusters of orthologous groups (COG) and **b** carbohydrate-active enzymes (CAZy) functional profiles. The groups (winter, summer) represent the dissimilarity among samples from the same season, while the “between” group quantifies the dissimilarity between the summer and winter sample groups, highlighting the seasonal difference in community composition. The Bray-Curtis distance plots confirm the clustering patterns observed in the principal coordinate analysis (PCoA)

The beta diversity analysis, which assessed differences in microbial functional profiles (COG) between seasons, revealed no significant seasonal structuring. Principal coordinate analysis (PCoA) of the COG profiles displayed a substantial overlap between winter and summer samples (Fig. 6a). The two principal coordinates explained a total of 45.7% of the variance in the data (PCoA1: 32.8%, PCoA2: 12.9%). The distribution of samples within the ordination space was not separated by season, which was supported by an ANOSIM test, as the seasonal groups did not differ (*R* = -0.002, *p* = 0.424).

Consistent with the PCoA results, the analysis of Bray-Curtis dissimilarity further confirmed the lack of distinct seasonal groupings. The median dissimilarity between samples from the same season (within-group distance) was comparable to the median dissimilarity between samples from different seasons (between-group distance), which indicated that the functional variation within each season was as great as the variation between winter and summer.

#### CAZy functional beta diversity

The beta diversity of carbohydrate-active enzyme (CAZy) profiles was analyzed to assess structural differences between winter and summer microbial communities. Differentiation in the functional potential for carbohydrate metabolism did not differ between seasons. Principal coordinate analysis (PCoA) revealed a substantial overlap between winter and summer within the ordination space (Fig. 6b). The two principal coordinates together explained 44.2% of the total variance in the CAZy profiles, with PCoA1 accounting for 29.7% and PCoA2 for 14.5%. Despite this explanatory power, the samples did not cluster by season and was supported by the ANOSIM test, which did not differ between seasonal groups (*R* = -0.045, *p* = 0.689).

The analysis of Bray-Curtis dissimilarity supported the PCoA findings. The median dissimilarity between samples from different seasons (between-group distance) was not greater than the median dissimilarity among samples within the same season (within-group distance for winter and summer), which indicated that the compositional variation in CAZy profiles between seasons was not distinct from the natural variation observed within each season.

### KEGG functional beta diversity

**Fig. 7:**
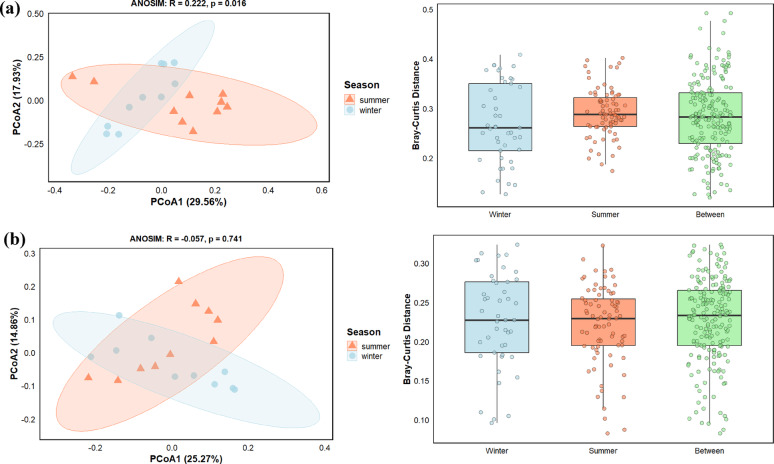
Seasonal beta diversity of microbial communities based on **a** Kyoto encyclopaedia of genes and genomes (KEGG) and **b** non-redundant (NR) protein databases. Principal coordinate analysis (PCoA) and Bray-Curtis dissimilarity plots display clustering of winter and summer samples

In contrast to the COG and CAZy profiles, the overall metabolic potential of the microbial community, based on KEGG orthologs, exhibited a significant seasonal shift. Principal coordinate analysis (PCoA) of the KEGG profiles revealed a noticeable separation between winter and summer samples along the two principal coordinates (Fig. 7a). These coordinates collectively explained 47.4% of the total variance, with PCoA1 contributing 29.5% and PCoA2 contributing 17.9%. The visual separation was validated by an ANOSIM test, which confirmed a significant, albeit weak, dissimilarity between the seasonal groups (*R* = 0.222, *p* = 0.016).

This seasonal pattern was further supported by the Bray-Curtis dissimilarity analysis. The median distance between samples from different seasons (between-groups) was markedly higher than the median distances among samples within the same season (within-group for winter and summer), which indicated that the differences in metabolic gene profiles between winter and summer were greater than the inherent variation within each season.

#### Beta diversity of taxonomic profile (NR)

The beta diversity of the microbial taxonomic composition, based on NR profiles, was analyzed to assess community structural differences between seasons. Principal coordinate analysis (PCoA) revealed extensive overlap between winter and summer samples within the ordination space (Fig. 7b). The two principal coordinates together explained 40% of the total variance in taxonomic profiles, with PCoA1 accounting for 25.2% and PCoA2 for 14.8%. Despite this considerable explanatory power, separation by season was not observed. An ANOSIM test confirmed the lack of a seasonal effect (*R* = -0.057, *p* = 0.741).

This finding was further supported by the Bray-Curtis dissimilarity analysis. The median dissimilarity between samples from different seasons was not greater than the median dissimilarity among samples within the same season, which indicated that the taxonomic compositional variation between winter and summer was negligible compared to the natural variation within each season.

### Seasonal impact on taxon functional composition

#### COG functional composition between seasons

**Fig. 8:**
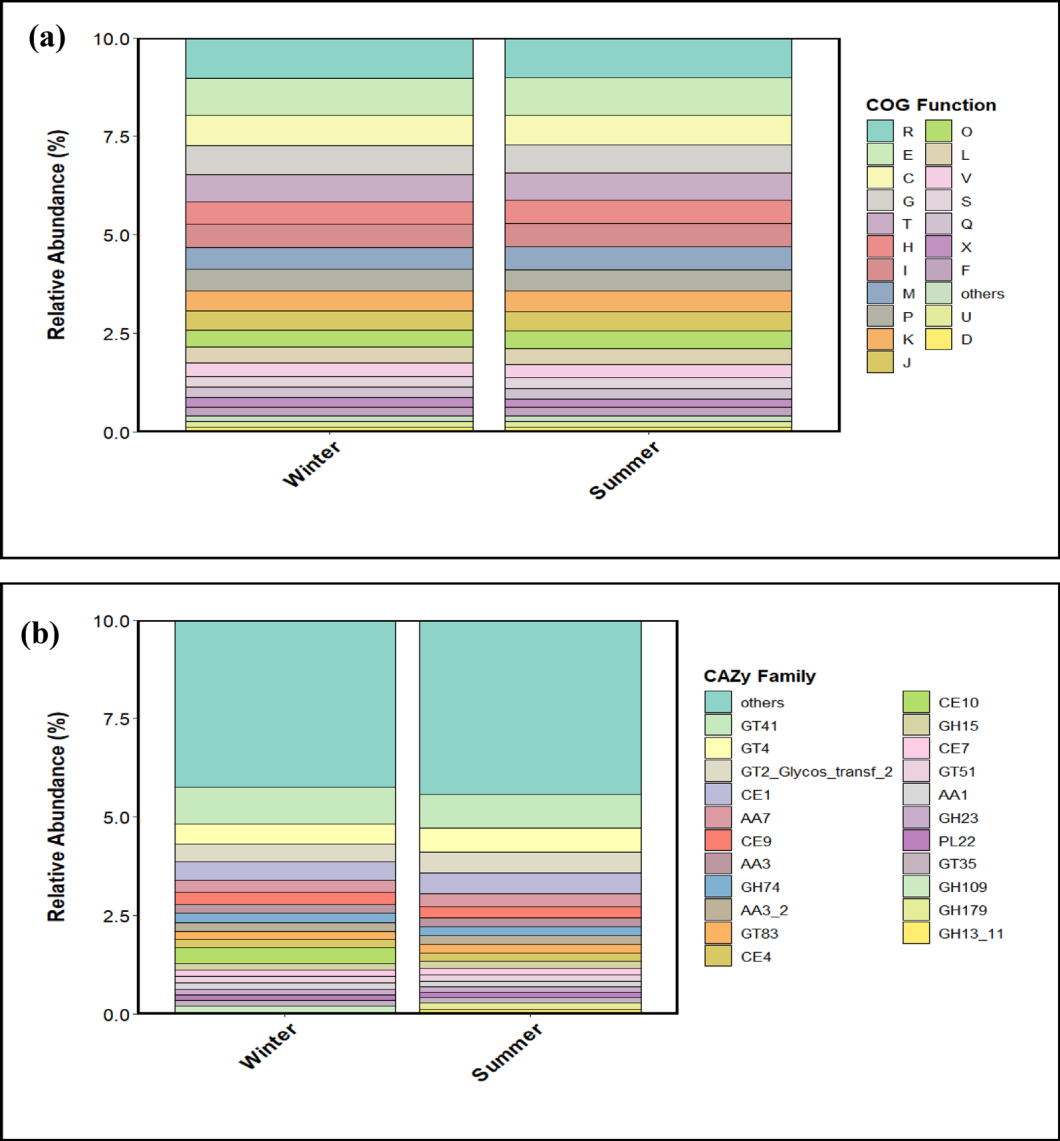
Stacked bar plots illustrating functional community abundances between seasons **a** Clusters of orthologous groups (COG) functional community abundance, R: General function prediction only, E: Amino acid transport and metabolism, C: Energy production and conversion, G: Carbohydrate transport and metabolism: Signal transduction mechanisms, H: Coenzyme transport and metabolism, I: Lipid transport and metabolism, P: Inorganic ion transport and metabolism, M: Cell wall/membrane/envelope biogenesis, K: Transcription, J: Translation, ribosomal structure and biogenesis, O: Posttranslational modification, protein turnover, chaperones, L:Replication, recombination and repair, V: Defence mechanisms, Q: Secondary metabolites biosynthesis, transport and catabolism, S: Function unknown, X: Mobilome: prophages, transposons, F: Nucleotide transport and metabolism, U: Intracellular trafficking, secretion, and vesicular transport, D: Cell cycle control, cell division, chromosome partitioning. **b** Carbohydrate active enzymes (CAZy) enzyme families

Seasonal analysis of clusters of orthologous groups (COG) functional category composition displayed minor fluctuations in relative abundances, with no consistent seasonal patterns. The dominant COG categories between seasons included R (general function prediction), E (amino acid transport and metabolism), C (energy production and conversion), and G (carbohydrate transport and metabolism), collectively accounting for most of the functional potential in microbial communities. Category R was the most abundant (0.099) between seasons, followed by E (0.087), C (0.075), and G (0.073). No difference (*p* > 0.05) in COG category abundances between seasons suggests functional redundancy, where diverse microbial taxa perform similar metabolic functions, and, thus, maintain ecosystem processes despite seasonal environmental changes. The high abundance of generalist functions like R supports essential roles in microbial survival and adaptation between seasons (Fig. 8a).

#### CAZy functional composition between seasons

Seasonal analysis of CAZy (carbohydrate-active enzymes) family composition revealed minor fluctuations in relative abundances of dominant families, with no consistent seasonal patterns. The dominant CAZy families between seasons included GT41 (glycosyltransferase family 41), GT4 (glycosyltransferase family 4), GT2_glycos_transf_2 (glycosyltransferase family 2), and CE1 (carbohydrate esterase family 1), collectively accounting for most of the carbohydrate-active enzymatic potential in microbial communities. GT41 was the most abundant (0.094) between seasons, followed by GT4 (0.060), GT2_glycos_transf_2 (0.052), and CE1 (0.05), highlighting the importance of carbohydrate esterification. Other families such as GT4 and GT2_glycos_transf_2 exhibited minimal seasonal variation. The lack of significant difference (*p* > 0.05) in CAZy family abundances between seasons suggested functional redundancy, where diverse microbial taxa performed similar carbohydrate-active functions, maintaining ecosystem processes despite seasonal environmental changes. The high abundance of generalist families such as GT41 supports their essential role in microbial survival and adaptation between seasons (Fig. 8b).

#### KEGG level 3 functional composition between seasons

Seasonal comparisons of KEGG level 3 pathways displayed minor variations in relative abundances, with no consistent seasonal trends. Metabolic pathways were the most abundant (0.168) followed by biosynthesis of secondary metabolites (0.070), microbial metabolism in diverse environments (0.058), and two-component systems (0.027), underscoring the role of signal transduction. Seasonal fluctuations were minimal, and no difference (*p* > 0.05) was observed in pathway abundances between seasons, which suggested functional redundancy within microbial communities, and, thus, different taxa performed similar metabolic functions, ensuring stability in ecosystem processes across seasonal changes. The high abundances of generalist pathways, such as metabolic pathways, further support their critical role in microbial adaptation and survival between seasons (Fig. S6a).

#### Microbial community composition between seasons

Seasonal analysis of microbial community composition revealed fluctuations in the relative abundances of dominant phyla, but no consistent seasonal patterns. *Actinomycetota* was the most abundant (0.361), followed by *Pseudomonadota* (0.256) and *Acidobacteriota* (0.129). Other notable phyla included *Verrucomicrobiota*, *Candidatus*_*Rokubacteria*, and *Chloroflexota*. Despite these differences, microbial community composition did not differ (*p* > 0.05) between seasons, indicating resilience to seasonal changes in environmental conditions. This stability suggests that microbial community structure supports consistent ecosystem functions between seasons, likely due to the ability of diverse taxa to maintain similar roles (Fig. S6b).

#### Seasonal drivers of the ecosystem: structural equation model (SEM)

**Fig. 9:**
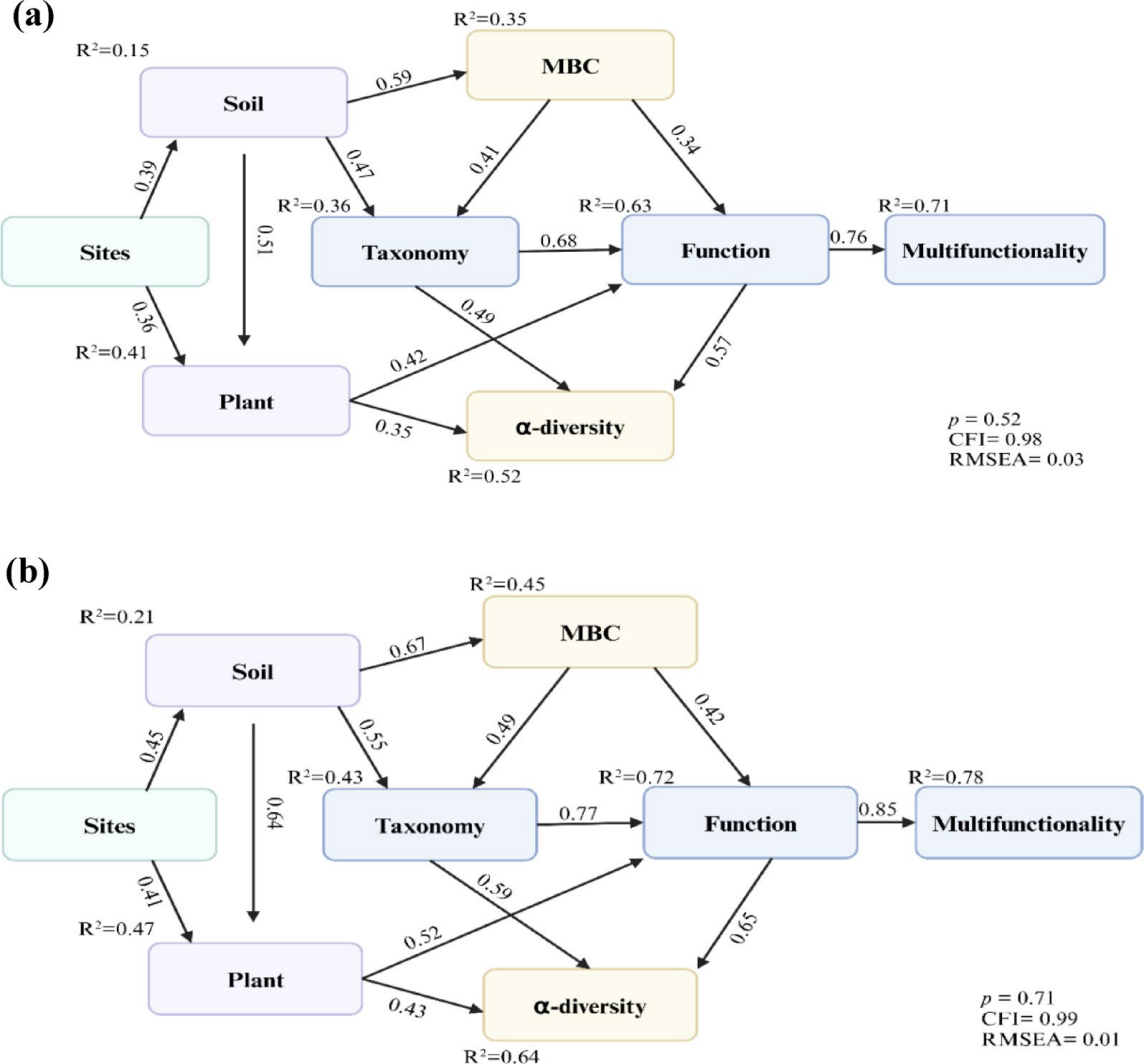
Structural equation models (SEM) (**a** winter; **b** summer) display relationships among soil properties, microbial α-diversity, and ecosystem multifunctionality. Explanatory power (R^2^ = 0.21–0.78) was stronger in summer than winter (R^2^ = 0.15–0.71), with microbial diversity being the highest predictive variable for multifunctionality (summer: R^2^ = 0.78; winter: R^2^ = 0.71).The values on the arrows are the standardized path coefficients and R^2^ is the proportion of variance explained for the dependent variable. Goodness of fit statistics for each model are presented

Structural equation models (SEMs) revealed distinct seasonal patterns, with stronger relationships between microbial α-diversity (summer R^2^=0.64; winter R^2^=0.52) and microbial biomass carbon (summer R^2^=0.45; winter R^2^=0.35) with ecosystem functioning in summer than in winter, as presented by comparative path coefficients in Table 3. Taxonomic (summer R^2^=0.43; winter R^2^=0.36) and functional diversities (summer R^2^=0.72; winter R^2^=0.63) were linked strongly to multifunctionality in summer, but weaker soil-microbe interactions in winter. The goodness-of-fit for both SEMs (Fig. 9a, b) was evaluated using multiple indices. The non-significant Chi-square (*p* > 0.05) for both models indicated an acceptable fit between the model structures and the observed data. The Chi-square combined with the comparative fit index (CFI) and the root mean square error of approximation (RMSEA) (summer: CFI = 0.99, RMSEA = 0.01; winter: CFI = 0.98, RMSEA = 0.03) confirmed a robust fit. This validation enabled us to confidently conclude that there are distinct and robust seasonal dynamics in the microbial-ecosystem relationships.

**Table 3:** Comparison of standardized path coefficients from the structural equation models for winter and summer seasons

Causal paths	Winter coefficients	Summer coefficients	Interpretation of consistency
Sites-soil	0.39	0.45	Consistent, slightly stronger in summer.
Sites-plant	0.36	0.41	Consistent, slightly stronger in summer.
Soil-plant	0.51	0.64	Consistent, moderately stronger in summer.
Soil-taxonomy	0.47	0.55	Consistent, moderately stronger in summer.
Soil-MBC	0.59	0.67	Consistent, moderately stronger in summer.
MBC-taxonomy	0.41	0.49	Consistent, moderately stronger in summer.
MBC-function	0.34	0.42	Consistent, moderately stronger in summer.
Plant-alpha diversity	0.35	0.43	Consistent, moderately stronger in summer.
Plant-function	0.42	0.52	Consistent, moderately stronger in summer.
Taxonomy-alpha diversity	0.49	0.59	Consistent, moderately stronger in summer.
Taxonomy-function	0.68	0.77	Consistently dominant, stronger in summer.
Function-alpha diversity	0.57	0.65	Consistent, moderately stronger in summer.
Function-multifunctionality	0.76	0.85	Consistently dominant, stronger in summer.

All pathways show a consistent pattern of being numerically stronger in the summer, indicating a seasonal amplification of the ecological network

## Discussion

The western Himalayan forests of Azad Jammu and Kashmir (AJK), Pakistan, represent a unique and ecologically important region characterized by diverse forests and seasonal variations. Our data revealed clear seasonal shifts in plant communities, with higher species richness and diversity indices in summer than in winter. This trend aligns with findings from temperate and Himalayan ecosystems, where seasonal changes in temperature and precipitation are known to influence vegetation dynamics. For example, studies in the eastern Himalayas [30] and European temperate forests also reported greater species richness and diversity during warmer than cooler months due to favorable growth conditions [31]. The consistent positive correlation between the Shannon and Simpson diversity indices in both seasons reflected a synergistic relationship where greater species richness co-occurred with greater community evenness. Gu et al. [32] observed that increased resource availability in summer promoted greater species evenness in forest ecosystems. However, our finding of lower community maturity during summer than winter indicated that younger, less stable communities may dominate in warmer months. This could be explained by rapid growth and turnover of species that contrasted with studies in tropical forests, where community maturity remained relatively stable across seasons [33]. The decline in maturity in the present study reflected the impact of environmental stressors, such as increased herbivory or competition, which can disrupt the establishment of mature plant communities. These findings underscore the importance of seasonal monitoring to understand the long-term impacts of climate change on forest ecosystems, particularly in regions like the western Himalayas, where seasonal variations are pronounced.

Soil properties exhibited seasonal and depth-related responses, reflecting the influence of environmental conditions on soil processes. Soil pH was higher in summer than in winter, which suggests that elevated temperatures and greater microbial activity increased soil alkalinity. Previous studies in temperate forests reported that seasonal warming increased soil pH due to enhanced microbial mineralization of organic matter [34]. The decrease we observed in soil moisture content and SOC in summer resulted from plant development and microbial decomposition, which depleted soil resources. Similarly, in Mediterranean forests, summer droughts and increased evapotranspiration reduced soil moisture content and accelerated organic matter decomposition [35]. The consistent decrease in SOC and TN with depth emphasized the role of topsoil organic matter in maintaining soil fertility, as demonstrated by previous research in mountainous regions [36]. Our data on nutrient availability revealed an increase in AP and a decrease in AK during summer, despite the stable TP and decrease in TK. This pattern suggests that microbial activity and root exudates enhanced the mobilization of specific nutrient in warmer months. In addition, seasonal changes in temperature and soil moisture influenced nutrient cycling and microbial activity in forest soils [37]. This interpretation was further supported by our observation of higher CEC and MBC in summer than in winter, which points to a strong role of microbial communities in nutrient mobilization and soil health, as was observed in other forest ecosystems [38].

Microbial alpha diversity and Shannon and Chao diversity indices were higher in summer than in winter, which likely reflected the more favorable environmental conditions for microbial growth and activity in summer. Nottingham et al. [39], indicated that higher temperatures and increased soil moisture enhanced microbial diversity in temperate forests. The beta diversity analysis revealed a key distinction in the microbial community’s seasonal response while taxonomic profiles (NR) and functional potentials for general proteins (COG) and carbohydrate-active enzymes (CAZy) remained stable, the broader metabolic pathways (KEGG) exhibited significant seasonal variation.

The stability in taxonomy and specific functional categories in the present study suggests a resilient core community where functional redundancy buffers against seasonal changes. Different taxa perform the same critical roles, ensuring that core processes like carbohydrate metabolism remain constant despite environmental fluctuations, as observed in other soil systems [40].

In contrast, the significant shift we detected in KEGG profiles indicated that the community’s overall metabolic strategy adapted to seasonal conditions. This divergence likely arises because KEGG maps genes to integrated pathways (e.g., energy metabolism, signal transduction) that are influenced directly by seasonal factors like temperature and substrate availability. The same microbial taxa appear to modulate their genomic potential for these broader metabolic processes, a form of functional plasticity that enables the community to maintain structural stability while fine-tuning its collective metabolic output in response to changing environmental conditions [41].

The functional composition of microbial communities, as inferred from COG, CAZy, and KEGG pathways, displayed strong consistency between seasons. The dominance of general functions, such as metabolic pathways and carbohydrate-active enzymes indicated functional redundancy within microbial communities. These enhance ecosystem resilience by ensuring that critical metabolic functions are maintained despite variations in environmental conditions and alterations in the microbial community. A previous study in forest ecosystems reported that functional redundancy stabilized nutrient cycling and organic matter decomposition under changing environmental conditions [42]. The lack of differences in functional composition between seasons in our study further supported the premise that microbial communities in the Western Himalayas are highly adaptable and resilient, as was observed in high-altitude ecosystems [43].

The increase we observed in microbial α-diversity and functional activity in summer aligned with findings from alpine soil studies [44], which reported that elevated temperatures promoted microbial metabolic rates and diversity. Our findings of stronger soil-microbe decoupling in summer than in winter in Himalayan forests indicated that reduced moisture availability during warm periods weakened microbial-SOC relationships [45]. The winter patterns of microbial dormancy and carbon stabilization supported the cold storage hypothesis proposed by Manzoni et al. [46] that low temperature preserves microbial biomass and organic matter. However, our findings that multifunctionality remained strongly microbial-linked in both seasons contrasted with some boreal studies [47], possibly reflecting the unique nature of Himalayan forests. These comparisons highlight how our findings both confirm general ecological principles about seasonal microbial ecology while exhibiting ecosystem-specific adaptations to the Himalayan environment.

A limitation of this study was the use of composite samples, which precludes an analysis of fine-scale, within-site variability. This approach was chosen to obtain a representative, site-level microbial profile for high-cost metagenomic sequencing between seasons across multiple forest sites. Our gene-centric analysis did not involve the reconstruction of Metagenome-Assembled Genomes (MAGs), which limits insights into the genomic traits of specific microbial populations. Future studies incorporating replicated sampling designs would be valuable in quantifying this micro-variation and confirm the robustness of the broad-scale ecological patterns observed in the present study while MAG-based approaches could provide deeper genomic resolution of key taxa.

## Conclusions

Our study demonstrates that the western Himalayan forest ecosystem undergoes a profound seasonal reorganization, driven by distinct shifts in vegetation, soil properties, and microbial activity. A consistent pattern of heightened biotic activity occurred in summer, evidenced by higher plant diversity, microbial alpha diversity (richness and Shannon diversity index), and key soil properties such as pH and nutrient availability. Conversely, the winter season was characterized by higher plant community maturity and elevated soil moisture and organic carbon pools.

The evidence of robust ecosystem resilience underpinned by a stable microbial taxonomic structure and functional redundancy emerged as the most critical findings. Despite significant seasonal changes in microbial alpha diversity, the overall taxonomic composition (beta diversity) of the communities remained stable between seasons. Functionally, the core profiles for general processes (COG) and carbohydrate metabolism (CAZy) also did not shift seasonally. However, a significant change in the community’s overall metabolic potential (KEGG orthologs) was detected, which indicates that while the cast of microbial characters and their core toolkits are stable, the specific metabolic functions they prioritize change with season. This dynamism enables the ecosystem to maintain stability while displaying plasticity in adapting to seasonal conditions.

Structural equation models (SEM) confirmed this dynamism, revealing more pronounced and interconnected plant-soil-microbe relationships in summer, yet a system that maintains a stable taxonomic and functional foundation year-round. This combination of taxonomic stability and functional flexibility is a key mechanism in enabling these high-altitude ecosystems to persist amidst strong seasonal selective pressures.

## Supplementary Information

Below is the link to the electronic supplementary material.

Supplementary Material 1

## Data Availability

The raw metagenomic sequencing data in this study are deposited in the China National Centre for Bioinformation (CNCB) under Bio Project accession number CRA027991. The data are publicly accessible via the CNCB repository (https://ngdc.cncb.ac.cn/gsa/browse/CRA027991).

## Declarations

### Authors’ details

^1^School of Life Sciences, Lanzhou University, Lanzhou 730000, China.

^2^State Key Laboratory of Herbage Improvement and Grassland Agro-ecosystems, College of Ecology, Lanzhou University, Lanzhou 730000, China.

^3^Department of Microbiology, Balochistan University of Information Technology, Engineering and Management Sciences, Quetta, Pakistan.

^4^Department of Plant Sciences, Quaid-i-Azam University Islamabad, Pakistan.

^5^Desert Animal Adaptations and Husbandry, Wyler Department of Dryland Agriculture, Blaustein Institutes for Desert Research, Ben-Gurion University of the Negev, Beer Sheva, 8410500, Israel.

^6^Rangeland Research Institute, National Agricultural Research Center, Islamabad 44000, Pakistan.
