# Supplementary material for: Seasonal shifts in vegetation, soil properties, and microbial communities in Western Himalayan forests
Source: Environ Microbiome. 2026 Jan 6;21:22. doi: 10.1186/s40793-025-00842-y (PMC12871016; doi:10.1186/s40793-025-00842-y)
Supplement: Supplementary file 1 — Supplementary Material 1 [file 40793_2025_842_MOESM1_ESM.docx]

**Supplementary material**

**Part of methodology**

**2.3.1 Soil EC and pH**

15 g of sieved soil were added to 30 mL distilled water (soil: water = 1:2, w/v), mixed, and shaken for 30 minutes. Soil EC and pH of the filtrate were determined with a digital EC meter (EC/TDS/Salinity Meter with ATC - HI2300, Hanna Instruments, Woonsocket, RI, USA) and pH meter (Lab pH meter 7110, WTW, Werkstätten, Germany), respectively [1].

**2.3.2 Soil moisture content**

10 g of wet soil sample were oven-dried in a crucible at 105˚C for 24 h. The samples were cooled in a desiccator and weighed. Soil moisture was determined following Dane and Topp [1].

$Soil moisture\left( \% \right)=\frac{(Wet soil weight-Dry soil weight)}{Wet soil weight}\times100$

**2.3.3 Soil bulk density**

Soil bulk density (BD) is the ratio of soil mass (oven-dry mass) to bulk volume expressed in g/cm^3^. Soil samples were collected in 100 cm^3^ volume metal rings and placed in polyethylene bags to avoid moisture loss. The oven-dry mass of the soil at 105˚C after 24 hours was used to calculate bulk density following Jones [2].

$BD(g/cm3)=\frac{W}{V}$

where W = dry mass of soil in the metal ring; V = volume of the metal ring.

**2.3.4 Soil texture**

Soil texture was determined by the hydrometer method in which a dispersing solution was prepared by dissolving 40 g sodium hexametaphosphate [(NaPO3)_13_] and 10 g sodium carbonate (Na_2_CO_3_) in distilled water to 1 L. A 40 g sieved and dried soil sample was added to 60 ml of dispersing solution, covered, and left overnight [3]. The next day, the content in the beaker was transferred to a soil stirring cup filled with 60 mL of water and stirred for five minutes. The suspension was transferred to a cylinder, bringing the volume to 1 L with deionized water, stirred for five minutes, and a hydrometer reading (HR_1_) was taken [3]. The solution was left for four hours, and a second hydrometer reading (HR_2_) was taken. Soil clay, silt, and sand were calculated as outlined by Hasan and Abuel-Naga [3].

$Clay+Silt\left( \% \right)=\frac{HR1-(T1C)}{wt. of soil}\times100$

$$Clay\left( \% \right)=\frac{HR2-(T2C)}{wt. of soil}\times100$$

$$Sand\left( \% \right)=100-(clay+silt)$$

$$Silt\left( \% \right)=\left( clay+silt \right)-clay$$

**2.3.5 Soil organic carbon**

A 0.5 g air-dried, sieved soil sample was placed in a 500 mL titration flask, and 10 mL 1 N potassium dichromate and 20 mL concentrated sulphuric acid were added, mixed, and left for 30 minutes. Approximately 200 mL distilled water, 10 mL orthophosphoric acid (H_3_PO_4_) and ten drops of diphenylamine indicator were added and mixed. The solution was titrated against 0.5 M ferrous ammonium sulphate solution until the colour changed from violet-blue to green. Soil organic matter and soil organic carbon were determined using the equations listed in Gerenfes et al. [4].

$$Organic matter\left( \% \right)=\frac{(Vblank-Vsample)\times0.5\times0.69}{wt.of sample}$$

$$Organic carbon\left( \% \right)=\frac{Organic matter}{1.724}$$

V_blank_ = volume of (NH_4_) 2SO_4_.FeSO_4_.6H2O solution required to titrate the blank (mL)

V_sample_ = volume of (NH_4_) 2SO_4_.FeSO_4_.6H2O solution required to titrate the sample (mL)

Wt = Weight of air-dry soil (g)

**2.3.6 Soil total phosphorus, potassium, and micronutrients measurements**

A 0.5 g air-dried, sieved soil sample was placed in a 250 mL conical flask and a nitric acid: hydrochloric acid mixture (2:1 v/v) was added for digestion and placed on a hot plate at 250 ˚C until white fumes appeared. Twenty mL distilled water were added to the cooled digested sample, filtered into a storage bottle, and distilled water was added to 50 mL [5]. The extract was then prepared according to the elements to be measured. Total potassium, total phosphorus and micronutrients (Fe, Cu, Mn, Zn) were measured using a flame photometer (Pg Instrument, Leicestershire, UK), spectrophotometer (L6/L6S Split Beam UV-VIS Spectrophotometer, Guangzhou, China) and atomic absorption spectrometer (SpectrAA 220FS, Palo Alto, CA, USA), respectively [5].

**2.3.7 Soil total nitrogen**

A 1 g air-dried, sieved soil sample was digested by sulphuric acid, and N content was determined by an automatic Kjeldahl nitrogen analyser (KJEL-AUTO, MRK, VELP Scientifica, Usmate, Italy) [6].

**2.3.8 Soil available phosphorus and potassium**

The ammonium bicarbonate diethylene triamine penta acetic acid (ABDTPA) method was used for soil extraction. The extraction solution was prepared by adding 1.97 g diethylene triamine penta acetic acid (DTPA) to 800 mL distilled water. Then 2 mL ammonium hydroxide (NH_4_OH): deionized water (1:1; v/v) were added to facilitate dissolution and to prevent effervescence when bicarbonate was added. The prepared solution contained 0.005 M DTPA. When most of the DTPA was dissolved, 79.06 g ammonium bicarbonate (NH_4_HCO_3_) were added, stirred gently, the pH was adjusted to 7.6 with NH_4_OH and brought to a volume of 1 L with distilled water. Thirty mL extraction solution were added to 15 g dried, sieved soil, shaken for 30 min, and the suspension was filtered by Whatman No. 42 filter paper. The extract was then prepared for phosphorus and potassium determination and was measured using a spectrophotometer (L6/L6S Split Beam UV-VIS Spectrophotometer, Guangzhou, China) and flame photometer (Pg Instrument, Leicestershire, UK), respectively [7].

**2.3.9 Cation exchange capacity**

4 g of air-dried, sieved soil were placed in a 40 mL centrifuge tube, and 33 mL of 1 N sodium acetate trihydrate solution were added. The cap was tightened on the tube and was shaken for five minutes. The cap was removed, the tube was centrifuged at 1006 g for five minutes and the clear supernatant was decanted and washed with sodium acetate three times, and then washed with 33 mL 95% ethanol, shaken, centrifuged and the clear supernatant was decanted. The adsorbed sodium was replaced with 1 N ammonium acetate solution, washed three times with 1 N ammonium acetate solution, shaken for five minutes, centrifuged, and the clear supernatant was stored in a 100 mL volumetric flask. After three washes, 1 N ammonium acetate solution was added to a volume of 100 mL. The samples were read by a flame photometer against sodium standards, and values were calculated as:

$$CEC\left( \frac{meq}{100g} \right)=\frac{meq\times V\times100}{Na\times wt.\times1000}$$

V = total volume of the soil extract (mL)

Wt = weight of air-dry soil (g)

**2.3.10 Microbial biomass carbon (MBC)**

MBC was measured by the fumigation-extraction and titration method. A beaker with 10 g of soil was placed in a desiccator and fumigated by 30 mL of chloroform that had been purified with 5% H_2_SO_4_ and distilled water and placed in the desiccator. The desiccator was closed tightly, a vacuum was created, and samples were fumigated. Another soil sample was also placed in a desiccator, but without chloroform, and these samples were non-fumigated. Both desiccators were stored in the dark for 24 hours [8]. The samples were removed from the desiccators, transferred to 250 mL conical flasks, and 50 mL K_2_SO_4_ were added, shaken for one hour, and filtered with Whatman no. 42 filter paper. Then 4 mL of extract were placed in a 250 mL conical flask, 1 mL of 0.667M K_2_Cr_2_O_7_ and 5 mL of conc.H_2_SO_4_ were added, and then 2 to 3 drops of phenanthroline amine indicator were added and titrated against 0.033 M ferrous sulphate solution till the colour changed from green to red. MBC was calculated as [8].

$MBC\left( \% \right)=\frac{\left( blank-sample \right)\times Nof FeSO4\times0.003}{wt.of sample \times1250}$

**
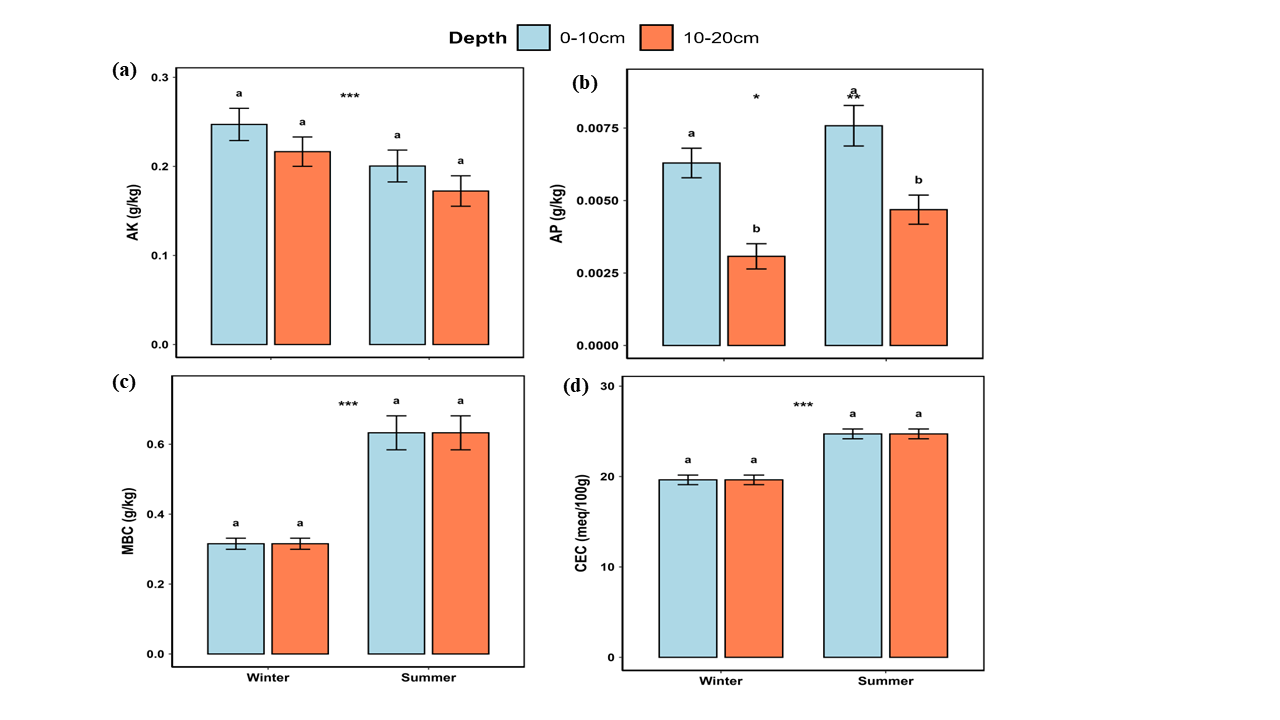
Supplementary figures**

**Figure S4.** Means ±SD of (a) available potassium (AK, g/kg), (b) available phosphorus (AP, g/kg), (c) microbial biomass carbon (MBC, g/kg) and (d) cation exchange capacity (CEC, meq/100g) in soil at 0-10 cm and 10-20 cm in winter and summer. Means with different letters within each season differ from each other (*p* < 0.05). Asterisks on shoulder lines indicate differences between seasons; **p* < 0.05, ***p* < 0.01, ****p* < 0.001.

**
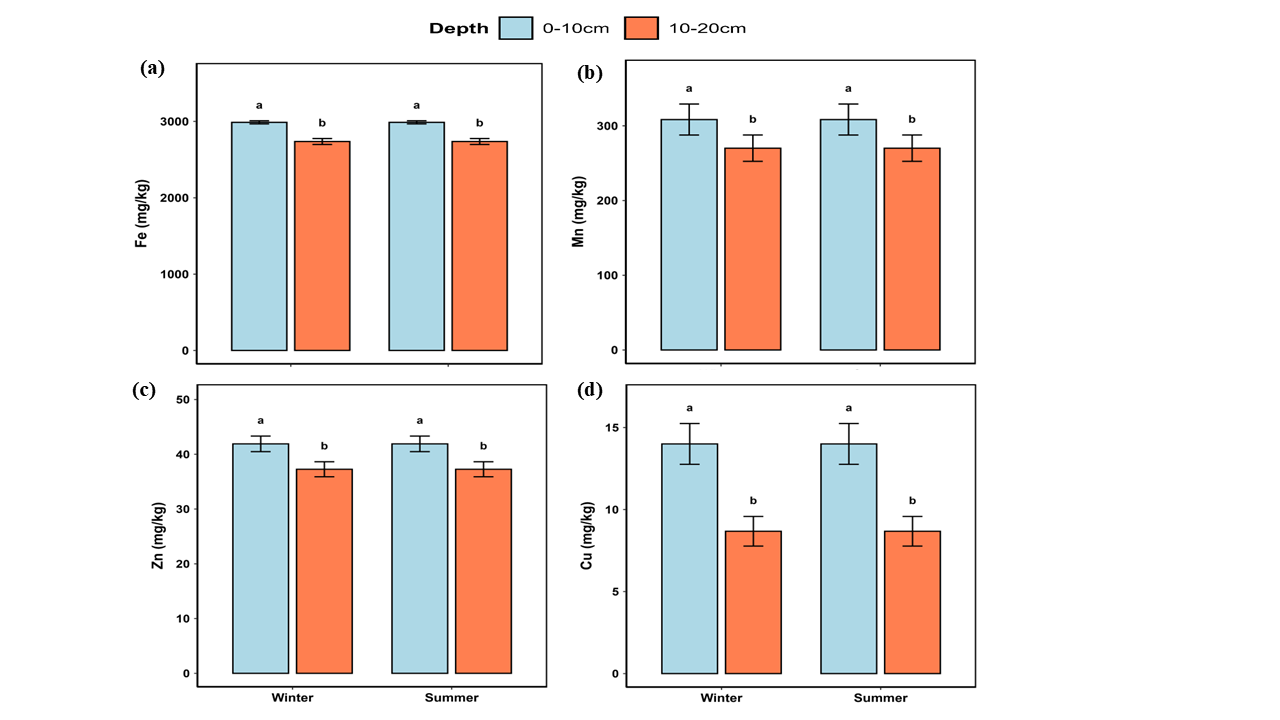
Figure S5.** Means ±SD (a) iron (Fe, mg/kg), (b) manganese (Mn, mg/kg), (c) zinc (Zn, mg/kg) and (d) copper (Cu, mg/kg) in soil at 0-10 cm and 10-20 cm. Means with different letters within each season differ from each other (*p* < 0.05). Asterisks on shoulder lines indicate differences between seasons; **p* < 0.05, ***p* < 0.01, ****p* < 0.001.


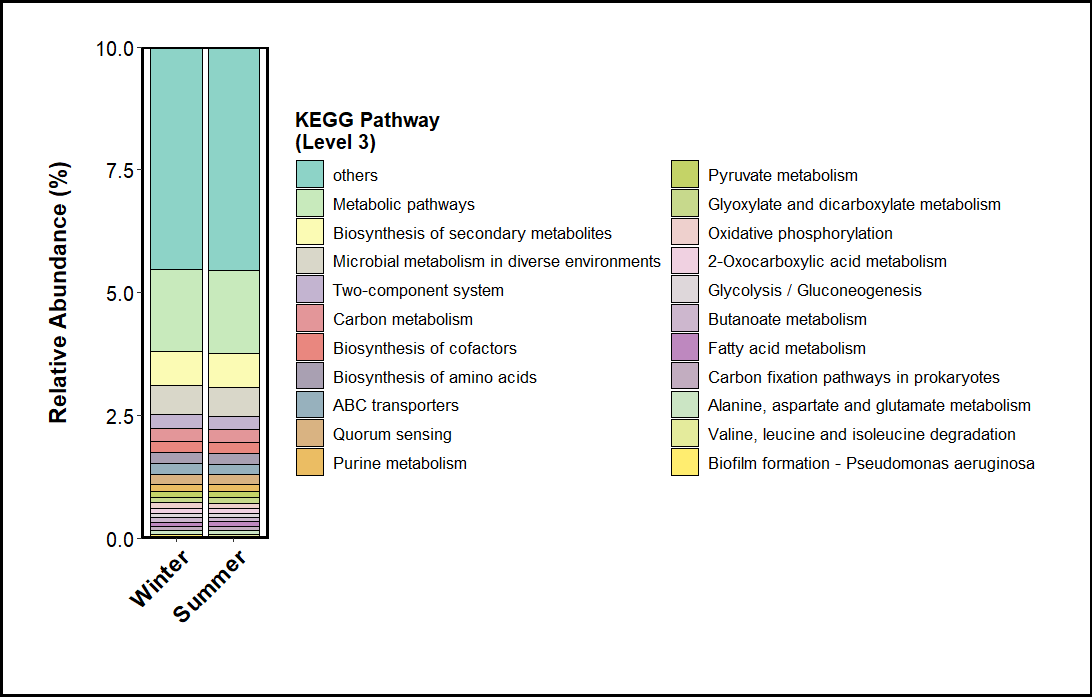

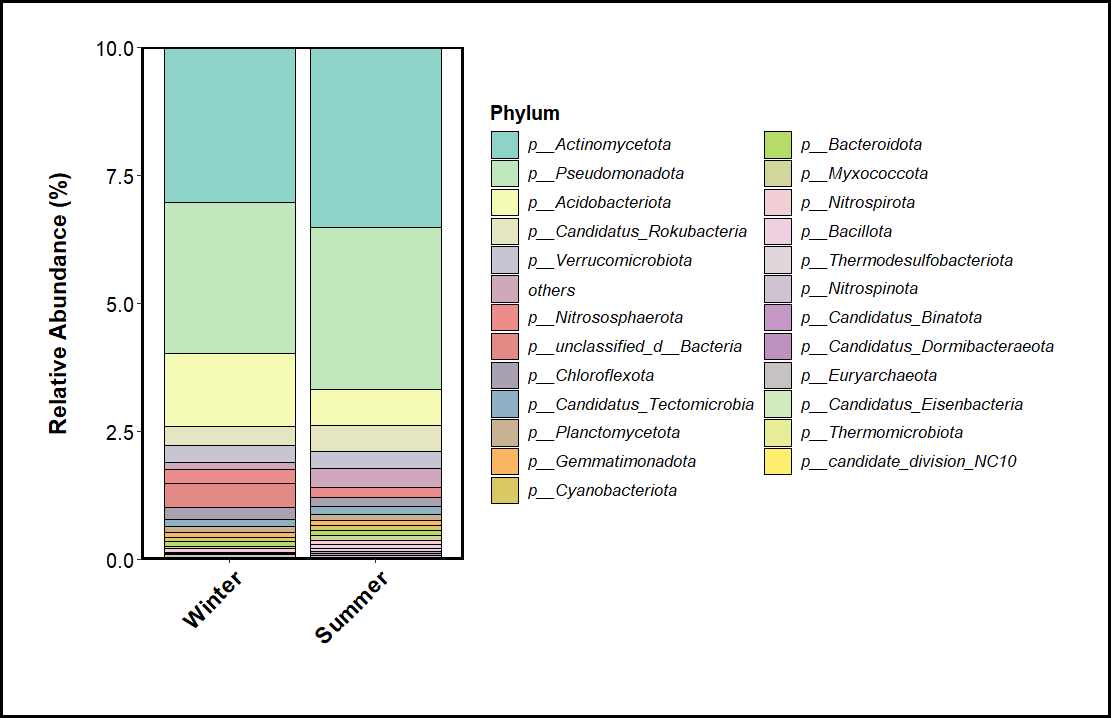


**(a)**

**(b)**

**Figure S6.** Stacked bar plots displaying community abundances between seasons; (a) Functional community abundance**,** Kyoto encyclopaedia of genes and genomes (KEGG) level 3 and (b) taxonomic community abundances at phylum level, Non-redundant (NR) between seasons.

**References**

1. Dane, J.H. and C.G. Topp, Methods of soil analysis, Part 4: Physical methods. 2020: John Wiley & Sons. [https://doi.org/10.1007/978-3-031-31458-2_4](https://protect.checkpoint.com/v2/r02/___https://doi.org/10.1007/978-3-031-31458-2_4___.YzJlOmJlbmd1cmlvbnVuaXZlcnNpdHlvZnRoZW5lZ2V2OmM6bzowZGM0Y2NhMTU5NTdiMmI4YmRmZThjYWNhM2QzMzk5Mjo3OjRjMmE6NzAyNmViY2M1ZWM3NzVkMGFmNjhmMjJiODEyMjcwMTZlNjQyN2ViZTFhODI1ZmE5ZDNjZmFhNGNiMDkzNDVlYzpwOlQ6Tg)

2. Jones Jr, J.B., Soil and Plant Analysis: Laboratory Registry for the United States and Canada. 2021: CRC Press.

3. Hasan, M.F. and H.J.M. Abuel-Naga, An Alternative Method of Obtaining the Particle Size Distribution of Soils by Electrical Conductivity. 2024. **14**(8): p. 804. [https://doi.org/10.3390/min14080804](https://protect.checkpoint.com/v2/r02/___https://doi.org/10.3390/min14080804___.YzJlOmJlbmd1cmlvbnVuaXZlcnNpdHlvZnRoZW5lZ2V2OmM6bzowZGM0Y2NhMTU5NTdiMmI4YmRmZThjYWNhM2QzMzk5Mjo3OmY2ZjY6YTViZTU1ZGM5MWE4NjM1OTY1MTIzZWIzYzZkNmZiNjEzYTQwOWFiOWUyNTA4OTE0YzU0OGY1OWFjMzBiNDJlMDpwOlQ6Tg)

4. Gerenfes, D., A. Giorgis, and G.J.I.J.H.F.S. Negasa, Comparison of organic matter determination methods in soil by loss on ignition and potassium dichromate method. Int. J. Hortic. Food Sci, 2022. **4**(1): p. 49-53. [https://doi.org/10.33545/26631067.2022.v4.i1a.85](https://protect.checkpoint.com/v2/r02/___https://doi.org/10.33545/26631067.2022.v4.i1a.85___.YzJlOmJlbmd1cmlvbnVuaXZlcnNpdHlvZnRoZW5lZ2V2OmM6bzowZGM0Y2NhMTU5NTdiMmI4YmRmZThjYWNhM2QzMzk5Mjo3OjEzOWU6Y2EwYzg4ODU4NGFlNzI3NDM1NjVjMGM5ZTc2ODYzN2FlNTQxZWI4NDUxNTc4NTM2ODRjZTk1NTk2ZWY0MTk2YzpwOlQ6Tg)

5. Sørensen, M.K., et al. Soil Analysis by Mobile Multinuclear NMR: Quantification of Phosphorus, Aluminum, and Sodium. Analytical Chemistry, 2024. **96**(43): p. 17086-17091. [https://doi.org/10.1021/acs.analchem.4c01333](https://protect.checkpoint.com/v2/r02/___https://doi.org/10.1021/acs.analchem.4c01333___.YzJlOmJlbmd1cmlvbnVuaXZlcnNpdHlvZnRoZW5lZ2V2OmM6bzowZGM0Y2NhMTU5NTdiMmI4YmRmZThjYWNhM2QzMzk5Mjo3OmI1OGY6OTA3MGVkODUzNmU1NWMxNmEzZTFjMmJlMzNmYjhhMTkwOTU2NDc4MThiY2U5NzFhODk4YTljZmI4MDk2YjEyODpwOlQ6Tg)

6. Aguirre, J., The Kjeldahl Method, in The Kjeldahl Method: 140 Years. 2023, Springer. p. 53-78.

7. Syed, A.J., Evaluation of Different Extractants forEstimation of P, K, Ca, Mg & Micro-nutrient Cations in Soils ofKashmirValley. 2022, SKUAST Kashmir. [https://doi.org/10.1080/01904167.2018.1426019](https://protect.checkpoint.com/v2/r02/___https://doi.org/10.1080/01904167.2018.1426019___.YzJlOmJlbmd1cmlvbnVuaXZlcnNpdHlvZnRoZW5lZ2V2OmM6bzowZGM0Y2NhMTU5NTdiMmI4YmRmZThjYWNhM2QzMzk5Mjo3OjUyOGI6Y2FmZjExNDdjNmUxYzljMTJmZTUzZjcwYTgwM2MwOWEyNGZlNzllMTRhNDZhYjdlY2NmMDhiNDk2MTZiNTBjNTpwOlQ6Tg)

8. Mori, T., et al., Is microbial biomass measurement by the chloroform fumigation extraction method biased by experimental addition of N and P? iForest-Biogeosciences and Forestry, 2021. 14(5): p. 408. [https://doi.org/10.3832/ifor3374-014](https://protect.checkpoint.com/v2/r02/___https://doi.org/10.3832/ifor3374-014___.YzJlOmJlbmd1cmlvbnVuaXZlcnNpdHlvZnRoZW5lZ2V2OmM6bzowZGM0Y2NhMTU5NTdiMmI4YmRmZThjYWNhM2QzMzk5Mjo3Ojk3OTM6YmU4ZGE0ZDVlNTcyN2RhMzhkN2JmZmViNTYwZjE2NDU5NmFmYTc2NzZmZDJlMGEyZGEwZTZkN2YyOTFmMDIzNTpwOlQ6Tg)
